# Cancer as a metabolic disease

**DOI:** 10.1186/1743-7075-7-7

**Published:** 2010-01-27

**Authors:** Thomas N Seyfried, Laura M Shelton

**Affiliations:** 1 Biology Department, Boston College, Chestnut Hill, MA 02467, USA

## Abstract

Emerging evidence indicates that impaired cellular energy metabolism is the defining characteristic of nearly all cancers regardless of cellular or tissue origin. In contrast to normal cells, which derive most of their usable energy from oxidative phosphorylation, most cancer cells become heavily dependent on substrate level phosphorylation to meet energy demands. Evidence is reviewed supporting a general hypothesis that genomic instability and essentially all hallmarks of cancer, including aerobic glycolysis (Warburg effect), can be linked to impaired mitochondrial function and energy metabolism. A view of cancer as primarily a metabolic disease will impact approaches to cancer management and prevention.

## Introduction

Cancer is a complex disease involving numerous tempo-spatial changes in cell physiology, which ultimately lead to malignant tumors. Abnormal cell growth (neoplasia) is the biological endpoint of the disease. Tumor cell invasion of surrounding tissues and distant organs is the primary cause of morbidity and mortality for most cancer patients. The biological process by which normal cells are transformed into malignant cancer cells has been the subject of a large research effort in the biomedical sciences for many decades. Despite this research effort, cures or long-term management strategies for metastatic cancer are as challenging today as they were 40 years ago when President Richard Nixon declared a war on cancer [1,2].

Confusion surrounds the origin of cancer. Contradictions and paradoxes have plagued the field [3-6]. Without a clear idea on cancer origins, it becomes difficult to formulate a clear strategy for effective management. Although very specific processes underlie malignant transformation, a large number of unspecific influences can initiate the disease including radiation, chemicals, viruses, inflammation, etc. Indeed, it appears that prolonged exposure to almost any provocative agent in the environment can potentially cause cancer [7,8]. That a very specific process could be initiated in very unspecific ways was considered "the oncogenic paradox" by Szent-Gyorgyi [8]. This paradox has remained largely unresolved [7].

In a landmark review, Hanahan and Weinberg suggested that six essential alterations in cell physiology could underlie malignant cell growth [6]. These six alterations were described as the hallmarks of nearly all cancers and included, 1) self-sufficiency in growth signals, 2) insensitivity to growth inhibitory (antigrowth) signals, 3) evasion of programmed cell death (apoptosis), 4) limitless replicative potential, 5) sustained vascularity (angiogenesis), and 6) tissue invasion and metastasis. Genome instability, leading to increased mutability, was considered the essential enabling characteristic for manifesting the six hallmarks [6]. However, the mutation rate for most genes is low making it unlikely that the numerous pathogenic mutations found in cancer cells would occur sporadically within a normal human lifespan [7]. This then created another paradox. If mutations are such rare events, then how is it possible that cancer cells express so many different types and kinds of mutations?

The loss of genomic "caretakers" or "guardians", involved in sensing and repairing DNA damage, was proposed to explain the increased mutability of tumor cells [7,9]. The loss of these caretaker systems would allow genomic instability thus enabling pre-malignant cells to reach the six essential hallmarks of cancer [6]. It has been difficult, however, to define with certainty the origin of pre-malignancy and the mechanisms by which the caretaker/guardian systems themselves are lost during the emergent malignant state [5,7].

In addition to the six recognized hallmarks of cancer, aerobic glycolysis or the Warburg effect is also a robust metabolic hallmark of most tumors [10-14]. Although no specific gene mutation or chromosomal abnormality is common to all cancers [7,15-17], nearly all cancers express aerobic glycolysis, regardless of their tissue or cellular origin. Aerobic glycolysis in cancer cells involves elevated glucose uptake with lactic acid production in the presence of oxygen. This metabolic phenotype is the basis for tumor imaging using labeled glucose analogues and has become an important diagnostic tool for cancer detection and management [18-20]. Genes for glycolysis are overexpressed in the majority of cancers examined [21,22].

The origin of the Warburg effect in tumor cells has been controversial. The discoverer of this phenomenon, Otto Warburg, initially proposed that aerobic glycolysis was an epiphenomenon of a more fundamental problem in cancer cell physiology, i.e., impaired or damaged respiration [23,24]. An increased glycolytic flux was viewed as an essential compensatory mechanism of energy production in order to maintain the viability of tumor cells. Although aerobic glycolysis and anaerobic glycolysis are similar in that lactic acid is produced under both situations, aerobic glycolysis can arise in tumor cells from damaged respiration whereas anaerobic glycolysis arises from the absence of oxygen. As oxygen will reduce anaerobic glycolysis and lactic acid production in most normal cells (Pasteur effect), the continued production of lactic acid in the presence of oxygen can represent an abnormal Pasteur effect. This is the situation in most tumor cells. Only those body cells able to increase glycolysis during intermittent respiratory damage were considered capable of forming cancers [24]. Cells unable to elevate glycolysis in response to respiratory insults, on the other hand, would perish due to energy failure. Cancer cells would therefore arise from normal body cells through a gradual and irreversible damage to their respiratory capacity. Aerobic glycolysis, arising from damaged respiration, is the single most common phenotype found in cancer.

Based on metabolic data collected from numerous animal and human tumor samples, Warburg proposed with considerable certainty and insight that irreversible damage to respiration was the prime cause of cancer [23-25]. Warburg's theory, however, was attacked as being too simplistic and not consistent with evidence of apparent normal respiratory function in some tumor cells [26-34]. The theory did not address the role of tumor-associated mutations, the phenomenon of metastasis, nor did it link the molecular mechanisms of uncontrolled cell growth directly to impaired respiration. Indeed, Warburg's biographer, Hans Krebs, mentioned that Warburg's idea on the primary cause of cancer, i.e., the replacement of respiration by fermentation (glycolysis), was only a symptom of cancer and not the cause [35]. The primary cause was assumed to be at the level of gene expression. The view of cancer as a metabolic disease was gradually displaced with the view of cancer as a genetic disease. While there is renewed interest in the energy metabolism of cancer cells, it is widely thought that the Warburg effect and the metabolic defects expressed in cancer cells arise primarily from genomic mutability selected during tumor progression [36-39]. Emerging evidence, however, questions the genetic origin of cancer and suggests that cancer is primarily a metabolic disease.

Our goal is to revisit the argument of tumor cell origin and to provide a general hypothesis that genomic mutability and essentially all hallmarks of cancer, including the Warburg effect, can be linked to impaired respiration and energy metabolism. In brief, damage to cellular respiration precedes and underlies the genome instability that accompanies tumor development. Once established, genome instability contributes to further respiratory impairment, genome mutability, and tumor progression. In other words, effects become causes. This hypothesis is based on evidence that nuclear genome integrity is largely dependent on mitochondrial energy homeostasis and that all cells require a constant level of useable energy to maintain viability. While Warburg recognized the centrality of impaired respiration in the origin of cancer, he did not link this phenomenon to what are now recognize as the hallmarks of cancer. We review evidence that make these linkages and expand Warburg's ideas on how impaired energy metabolism can be exploited for tumor management and prevention.

### Energetics of the living cell

In order for cells to remain viable and to perform their genetically programmed functions they must produce usable energy. This energy is commonly stored in ATP and is released during the hydrolysis of the terminal phosphate bond. This is generally referred to as the free energy of ATP hydrolysis [40-42]. The standard energy of ATP hydrolysis under physiological conditions is known as ΔG'_**ATP **_and is tightly regulated in all cells between -53 to -60 kJ/mol [43]. Most of this energy is used to power ionic membrane pumps [10,40]. In cells with functional mitochondria, this energy is derived mostly from oxidative phosphorylation where approximately 88% of total cellular energy is produced (about 28/32 total ATP molecules). The other approximate 12% of energy is produced about equally from substrate level phosphorylation through glycolysis in the cytoplasm and through the TCA cycle in the mitochondrial matrix (2 ATP molecules each). Veech and co-workers showed that the ΔG'_**ATP **_of cells was empirically formalized and measurable through the energies of ion distributions via the sodium pump and its linked transporters [42]. The energies of ion distributions were explained in terms of the Gibbs-Donnan equilibrium, which was essential for producing electrical, concentration, and pressure work.

A remarkable finding was the similarity of the ΔG'_**ATP **_among cells with widely differing resting membrane potentials and mechanisms of energy production. For example, the ΔG'_**ATP **_in heart, liver, and erythrocytes was approximately - 56 kJ/mol despite having very different electrical potentials of - 86, - 56, and - 6 mV, respectively [42]. Moreover, energy production in heart and liver, which contain many mitochondria, is largely through respiration, whereas energy production in the erythrocyte, which contains no nucleus or mitochondria, is entirely through glycolysis. Warburg also showed that the total energy production in quiescent kidney and liver cells was remarkably similar to that produced in proliferating cancer cells [24]. Despite the profound differences in resting potentials and in mechanisms of energy production among these disparate cell types, they all require a similar amount of total energy to remain viable.

The constancy of the ΔG'_**ATP **_of approximately -56 kJ/mol is fundamental to cellular homeostasis and its relationship to cancer cell energy is pivotal. The maintenance of the ΔG'_**ATP **_is the "end point" of both genetic and metabolic processes and any disturbance in this energy level will compromise cell function and viability [40]. Cells can die from either too little or too much energy. Too little energy will lead to cell death by either necrotic or apoptotic mechanisms, whereas over production of ATP, a polyanionic Donnan active material, will disrupt the Gibbs-Donnan equilibrium, alter the function of membrane pumps, and inhibit respiration and viability [42]. Glycolysis or glutaminolysis must increase in cells suffering mitochondrial impairment in order to maintain an adequate ΔG'_**ATP **_for viability. This fact was clearly illustrated in showing that total cellular energy production was essentially the same in respiration-normal and respiration-deficient fibroblasts [44].

In addition to its role in replenishing TCA cycle intermediates (anaplerosis), glutamine can also provide energy through stimulation of glycolysis in the cytoplasm and through substrate level phosphorylation in the TCA cycle (glutaminolysis) [45-49]. Energy obtained through substrate level phosphorylation in the TCA cycle can compensate for deficiencies in either glycolysis or oxidative phosphorylation [46,48,50], and can represent a major source of energy for the glutamine-dependent cancers. More energy is produced through substrate level phosphorylation in cancer cells than in normal cells, which produce most of their energy through oxidative phosphorylation. A major difference between normal cells and cancer cells is in the origin of the energy produced rather than in the amount of energy produced since approximately -56 kJ/mol is the amount of energy required for cell survival regardless of whether cells are quiescent or proliferating or are mostly glycolytic or respiratory. It is important to recognize, however, that a prolonged reliance on substrate level phosphorylation for energy production produces genome instability, cellular disorder, and increased entropy, i.e., characteristics of cancer [8,24].

### Mitochondrial function in cancer cells

Considerable controversy has surrounded the issue of mitochondrial function in cancer cells [18,29,30,33,34,51-57]. Sidney Weinhouse and Britton Chance initiated much of this controversy through their critical evaluation of the Warburg theory and the role of mitochondrial function [33,34]. Basically, Weinhouse felt that quantitatively and qualitatively normal carbon and electron transport could occur in cancer cells despite the presence of elevated glycolysis [33,34]. Weinhouse assumed that oxygen consumption and CO_2 _production were indicative of coupled respiration. However, excessive amounts of Donnan active material (ATP) would be produced if elevated glycolysis were expressed together with coupled respiration [42]. Accumulation of Donnan active material will induce cell swelling and produce a physiological state beyond the Gibbs-Donnan equilibrium. The occurrence of up-regulated glycolysis together with normal coupled respiration is incompatible with metabolic homeostasis and cell viability. Chance and Hess also argued against impaired respiration in cancer based on their spectrophotometric studies showing mostly normal electron transfer in ascites tumor cells [58]. These studies, however, failed to assess the level of ATP production as a consequence of normal electron transfer and did not exclude the possibility of elevated ATP production through TCA cycle substrate level phosphorylation. As discussed below, mitochondrial uncoupling can give the false impression of functional respiratory capacity.

Oxygen uptake and CO_2 _production can occur in mitochondria that are uncoupled and/or dysfunctional [24,59]. While reduced oxygen uptake can be indicative of reduced oxidative phosphorylation, increased oxygen uptake may or may not be indicative of increased oxidative phosphorylation and ATP production [59-62]. Ramanathan and co-workers showed that oxygen consumption was greater, but oxygen dependent (aerobic) ATP synthesis was less in cells with greater tumorigenic potential than in cells with lower tumorigenic potential [61]. These findings are consistent with mitochondrial uncoupling in tumor cells. It was for these types of observations in other systems that Warburg considered the phenomenon of aerobic glycolysis as too capricious to serve as a reliable indicator of respiratory status [24]. Heat production is also greater in poorly differentiated high glycolytic tumor cells than in differentiated low glycolytic cells [63]. Heat production is consistent with mitochondrial uncoupling in these highly tumorigenic cells. Although Burk, Schade, Colowick and others convincingly dispelled the main criticisms of the Warburg theory [55,57,64], citations to the older arguments for normal respiration in cancer cells persist in current discussions of the subject.

Besides glucose, glutamine can also serve as a major energy metabolite for some cancers [65-67]. Glutamine is often present in high concentrations in culture media and serum. Cell viability and growth can be maintained from energy generated through substrate level phosphorylation in the TCA cycle using glutamine as a substrate [47,48]. Energy obtained through this pathway could give the false impression of normal oxidative phosphorylation, as oxygen consumption and CO_2 _production can arise from glutaminolysis and uncoupled oxidative phosphorylation. Hence, evidence suggesting that mitochondrial function is normal in cancer cells should be considered with caution unless data are provided, which exclude substrate level phosphorylation through glutaminolysis or glycolysis as alternative sources of energy.

### Mitochondrial dysfunction in cancer cells

Numerous studies show that tumor mitochondria are structurally and functionally abnormal and incapable of generating normal levels of energy [10,60,61,68-74]. Recent evidence also shows that the *in vitro *growth environment alters the lipid composition of mitochondrial membranes and electron transport chain function [75]. Moreover, the mitochondrial lipid abnormalities induced from the *in vitro *growth environment are different from the lipid abnormalities found between normal tissue and tumors that are grown *in vivo*. It appears that the *in vitro *growth environment reduces Complex I activity and obscures the boundaries of the Crabtree and the Warburg effects. The Crabtree effect involves the inhibition of respiration by high levels of glucose [76,77], whereas the Warburg effect involves inhibition of respiration from impaired oxidative phosphorylation. While the Crabtree effect is reversible, the Warburg effect is largely irreversible. Similarities in mitochondrial lipids found between lung epidermoid carcinoma and fetal lung cells are also consistent with respiratory defects in tumor cells [78]. The bioenergetic capacity of mitochondria is dependent to a large extent on the content and composition of mitochondrial lipids.

Alterations in mitochondrial membrane lipids and especially the inner membrane enriched lipid, cardiolipin, disrupt the mitochondrial proton motive gradient (ΔΨ_m_) thus inducing protein-independent uncoupling with concomitant reduction in respiratory energy production [41,73,79-82]. Cancer cells contain abnormalities in cardiolipin content or composition, which are associated with electron transport abnormalities [73]. Cardiolipin is the only lipid synthesized almost exclusively in the mitochondria. Proteins of the electron transport chain evolved to function in close association with cardiolipin. Besides altering the function of most electron transport chain complexes including the F1-ATPase, abnormalities in cardiolipin content and composition can also inhibit uptake of ADP through the adenine nucleotide transporter thus altering the efficiency of oxidative phosphorylation [41,79-81,83]. Abnormalities in the content and composition of cardiolipin will also prevent oxidation of the coenzyme Q couple thus producing reactive oxygen species during tumor progression [73,84]. Increased ROS production can impair genome stability, tumor suppressor gene function, and control over cell proliferation [7,85]. Hence, abnormalities in CL can alter cancer cell respiration in numerous ways.

Cardiolipin abnormalities in cancer cells can arise from any number of unspecific influences to include damage from mutagens and carcinogens, radiation, low level hypoxia, inflammation, ROS, or from inherited mutations that alter mitochondrial energy homeostasis [73]. Considering the dynamic behavior of mitochondria involving regular fusions and fissions [86], abnormalities in mitochondrial lipid composition and especially of cardiolipin could be rapidly disseminated throughout the cellular mitochondrial network and could even be passed along to daughter cells somatically, through cytoplasmic inheritance.

Besides lipidomic evidence supporting the Warburg cancer theory [73], recent studies from Cuezva and colleagues also provide compelling proteomic evidence supporting the theory [21]. Their results showed a drop in the β-F1-ATPase/Hsp60 ratio concurrent with an upregulation of the glyceraldehyde-3-phosphate dehydrogenase potential in most common human tumors [72]. These and other observations indicate that the bioenergetic capacity of tumor cells is largely defective [87-89]. Viewed collectively, the bulk of the experimental evidence indicates that mitochondria structure and function is abnormal in cancer cells. Hence, mitochondrial dysfunction will cause cancer cells to rely more heavily than non-cancer cells on substrate level phosphorylation for energy production in order to maintain membrane pump function and cell viability.

### Linking genome instability to mitochondrial dysfunction

Is it genomic instability or is it impaired energy metabolism that is primarily responsible for the origin of cancer? This is more than an academic question, as the answer will impact approaches to cancer management and prevention. Metabolic studies in a variety of human cancers previously showed that that loss of mitochondrial function preceded the appearance of malignancy and aerobic glycolysis [90]. However, the general view over the last 50 years has been that gene mutations and chromosomal abnormalities underlie most aspects of tumor initiation and progression including the Warburg effect and impaired respiratory function. The gene theory of cancer would argue that mitochondrial dysfunction is an effect rather than a cause of cancer, whereas the metabolic impairment theory would argue the reverse. If gene mutations are the primary cause of cancer then the disease can be considered etiologically complicated requiring multiple solutions for management and prevention. This comes from findings that the numbers and types of mutations differ markedly among and within different types of tumors. If, on the other hand, impaired energy metabolism is primarily responsible for cancer, then most cancers can be considered a type of metabolic disease requiring fewer and less complicated solutions.

Although mitochondrial function and oxidative phosphorylation is impaired in tumor cells, it remains unclear how these impairments relate to carcinogenesis and to the large number of somatic mutations and chromosomal abnormalities found in tumors [7,15,91-93]. Most inherited "inborn errors of metabolism" do not specifically compromise mitochondrial function or cause cancer in mammals. There are some exceptions, however, as germ-line mutations in genes encoding proteins of the TCA cycle can increase risk to certain human cancers [94]. For example, risk for paraganglioma involves mutations in the succinate dehydrogenase gene, whereas risk for leiomyomatosis and renal cell carcinoma involves mutations in the fumarate hydratase (fumarase) gene [94-97]. These and similar mutations directly impair mitochondrial energy production leading to increased glycolysis and the Warburg effect [98]. Although rare inherited mutations in the p53 tumor suppressor gene can increase risk for some familial cancers of the Li Fraumeni syndrome [99], most p53 defects found in cancers are not inherited and appear to arise sporadically, as do the vast majority of cancer-associated mutations [6,7,100]. In general, cancer-causing germline mutations are rare and contribute to only about 5-7% of all cancers [5,7]. While germline mutations can cause a few cancers, most cancer mutations are somatic and will contribute more to the progression than to the origin of most cancers.

The cancer mutator phenotype was invoked to explain the large number of somatic mutations found in cancer, but mutations in the p53 caretaker gene are not expressed in all cancers nor does p53 deletion produce cancer in mice suggesting a more complicated involvement of this and other genome guardians in carcinogenesis [7,101-104]. While numerous genetic abnormalities have been described in most human cancers, no specific mutation is reliably diagnostic for any specific type of tumor [7,17,105]. On the other hand, few if any tumors are known, which express normal respiration.

### Retrograde response and genomic instability

As an alternative to the genome guardian hypothesis for the origin of somatic mutations, a persistent retrograde response can underlie the genomic instability and mutability of tumor cells. The retrograde (RTG) response is the general term for mitochondrial signaling and involves cellular responses to changes in the functional state of mitochondria [106-110]. Although the RTG response has been most studied in yeast, mitochondrial stress signaling is an analogous response in mammalian cells [110,111]. Expression of multiple nuclear genes controlling energy metabolism is profoundly altered following impairment in mitochondrial energy homeostasis [112,113]. Mitochondrial impairment can arise from abnormalities in mtDNA, the TCA cycle, the electron transport chain, or in the proton motive gradient (ΔΨ_m_) of the inner membrane. Any impairment in mitochondrial energy production can trigger an RTG response. The RTG response evolved in yeast to maintain cell viability following periodic disruption of mitochondrial ATP production [110,114]. This mostly involves an energy transition from oxidative phosphorylation to substrate level phosphorylation. Similar systems are also expressed in mammalian cells [110-113]. Prolonged or continued activation of the retrograde response, however, can have dire consequences on nuclear genome stability and function.

**Figure 1:**
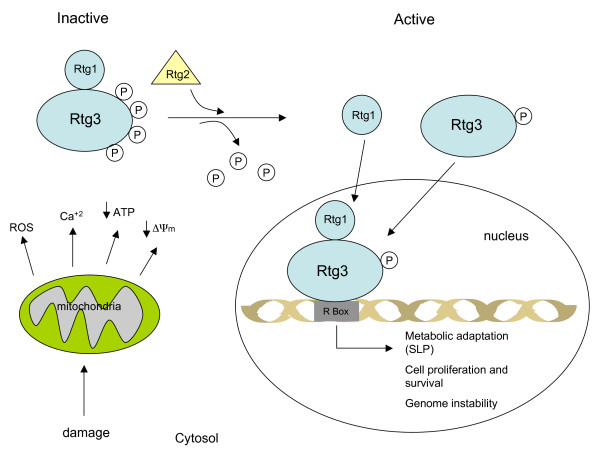
**Activation of the retrograde response (RTG) response in yeast cells**. The circled Ps are phosphate groups. SLP, (substrate level phosphorylation). See text for description of the RTG response.

Three main regulatory elements define the RTG response in yeast to include the Rtg2 signaling protein, and the Rtg1/Rtg-3 transcriptional factor complex (both are basic helix-loop-helix-leucine zippers) [110]. Rtg2 contains an N-terminal ATP binding motif that senses changes in mitochondrial ATP production. Rtg2 also regulates the function and cellular localization of the heterodimeric Rtg1/Rtg-3 complex (Figure 1). The RTG response is "off" in healthy cells with normal mitochondrial function. In the off state, the Rtg1/Rtg3 complex is sequestered in the cytoplasm with Rtg1 attached (dimerized) to a highly phosphorylated form of Rtg3 [110]. Besides its role in the cytoplasm as an energy sensor, Rtg2 also functions in the nucleus as a regulator of chromosomal integrity [115].

The RTG response is turned "on" following impairment in mitochondrial energy production. In the on state, cytoplasmic Rtg2 disengages the Rtg1/Rtg-3 complex through a dephosphorylation of Rtg3 [110]. The Rtg1 and Rtg3 proteins then individually enter the nucleus where Rtg3 binds to R box sites, Rtg1 reengages Rtg3, and transcription and signaling commences for multiple energy and anti-apoptotic related genes and proteins to include MYC, TOR, p53, Ras, CREB, NFkB, and CHOP [110,112,113,116-118]. The RTG response also involves the participation of multiple negative and positive regulators, which facilitate the bioenergetic transition from respiration to substrate level phosphorylation [110].

The primary role of the RTG response is to coordinate the synthesis of ATP through glycolysis alone or through a combination of glycolysis and glutaminolysis when respiratory function is impaired [110,111]. The RTG response would be essential for maintaining a stable ΔG'_**ATP **_for cell viability during periods when respiration is impaired. A prolonged RTG response, however, would leave the nuclear genome vulnerable to instability and mutability [112,117,119]. Mitochondrial dysfunction also increases levels of cytoplasmic calcium, the multi-drug resistance phenotype, production of reactive oxygen species, and abnormalities in iron-sulfur complexes, which together would further accelerate aberrant RTG signaling and genome mutability [85,106,107,110,111,120-122]. Chronic tissue inflammation could further damage mitochondria, which would accelerate these processes [123,124]. Considered collectively, these findings indicate that the integrity of the nuclear genome is dependent to a large extent on the functionality and energy production of the mitochondria.

### Similarities between yeast cells and mammalian cells to impaired respiration

Interesting analogies exist between yeast and mammalian cells for the physiological response to impaired respiration [76,112,117,125,126]. Mammalian cells increase expression of hypoxia-inducible factor-1a (HIF-1α) in response to transient hypoxia [127]. HIF-1α is rapidly degraded under normoxic conditions, but becomes stabilized under hypoxia. This is a conserved physiological response that evolved to protect mammalian mitochondria from hypoxic damage and to provide an alternative source of energy to respiration, as HIF-1α induces expression of pyruvate dehydrogenase kinase 1 and most major genes involved with glucose uptake, glycolysis, and lactic acid production [127]. HIF-1α expression is also elevated in most tumor cells whether or not hypoxia is present and could mediate in part aerobic glycolysis [20,28,98,128,129]. Although the mechanisms of HIF-1α stabilization under hypoxic conditions are well defined, the mechanisms by which HIF-1α is stabilized under aerobic or normoxic conditions are less clear [129,130].

HIF-1α is generally unstable in cells under normal aerobic conditions through its interaction with the von Hippel-Lindau (VHL) tumor suppressor protein, which facilitates HIF-1α hydroxylation, ubiquitination, and proteasomal degradation [28]. HIF-1α stabilization under aerobic conditions can be linked to mitochondrial dysfunction through abnormalities in calcium homeostasis, ROS generation, NFkB signaling, accumulation of TCA cycle metabolites (succinate and fumarate), and oncogenic viral infections [131-135]. It is not yet clear if genomic instability can arise through prolonged HIF-1α stabilization under aerobic conditions as would occur during tumor initiation and progression.

Besides HIF-1α function, the human MYC transcription factor also shows homology to the yeast Rtg3 transcription factor [112]. MYC is also a member of the basic, helix-loop-helix leucine zipper family of transcription factors as are RTG1 and RTG3. HIF-1α and MYC also up-regulate many of the same genes for glycolysis [136]. Hence, both HIF-1α and MYC share similarities with components of the yeast RTG system.

### Mitochondrial dysfunction and the mutator phenotype

Most human cancer cells display genome instability involving elevated mutation rates, gross chromosomal rearrangements, and alterations in chromosome number [15,17,100,137]. The recent studies of the Singh and the Jazwinski groups provide compelling evidence that mitochondrial dysfunction, operating largely through the RTG response (mitochondrial stress signaling), can underlie the mutator phenotype of tumor cells [71,113,115,117,138]. Chromosomal instability, expression of gene mutations, and the tumorigenic phenotype were significantly greater in human cells with mtDNA depletion than in cells with normal mtDNA. Mitochondrial dysfunction can also down-regulate expression of the apurinic/apyrimidinic endonuclease APE1. This is a redox-sensitive multifunctional endonuclease that regulates DNA transcription and repair [113,139]. APE1 down regulation will increase genomic mutability. Since gene expression is different in different tissues, it is expected that disturbed energy metabolism would produce different kinds of mutations in different types of cancers. Even different tumors within the same cancer type could appear to represent different diseases when evaluated at the genomic level. When evaluated at the metabolic level, however, most cancers and tumors are alike in expressing mitochondrial dysfunction and elevated substrate level phosphorylation. Emerging evidence suggests that mitochondrial dysfunction underlies the mutator phenotype of tumor cells.

Impaired mitochondrial function can induce abnormalities in tumor suppressor genes and oncogenes. For example, impaired mitochondrial function can induce abnormalities in p53 activation, while abnormalities in p53 expression and regulation can further impair mitochondrial function [85,103,113,116,140-143]. The function of the pRB tumor suppressor protein, which controls the cell cycle, is also sensitive to ROS production through the redox state of the cell [144]. Elevated expression of the *MYC *and *Ras *oncogenes can be linked to the requirements of substrate level phosphorylation to maintain tumor cell viability. Hence, the numerous gene defects found in various cancers can arise as secondary consequences of mitochondrial dysfunction.

Calcium homeostasis is also dependent on mitochondrial function [110]. It appears that calcium homeostasis is essential for the fidelity of mitosis to include spindle assembly, sister chromosome separation, and cytokinesis [145-150]. Disturbances in cytoplasmic calcium homeostasis, arising as a consequence of mitochondrial dysfunction [111], could contribute to abnormalities in chromosomal segregation during mitosis. These findings suggest that the numerous chromosomal abnormalities found in cancer cells can arise as a consequence of mitochondrial damage.

Recent studies in yeast indicate that damage to the inner mitochondrial membrane potential (ΔΨ_m_) following loss of mtDNA alters the function of several nuclear iron-sulfur-dependent DNA repair enzymes involving the Rad3 helicase, the Pri2 primase, and the Ntg2 glycase [107]. Abnormalities in these DNA repair enzymes contribute to the loss of heterozygosity (LOH) phenotype in specific genes of the affected yeast cells. These findings indicate that LOH, which is commonly observed in many genes of cancer cells [100], can also be linked to mitochondrial dysfunction. Considered collectively, these observations suggest that the bulk of the genetic abnormalities found in cancer cells, ranging from point mutations to gross chromosomal rearrangements, can arise following damage to the structure and function of mitochondria.

Impairment of mitochondrial function can occur following prolonged injury or irritation to tissues including disruption of morphogenetic fields [123,151]. This tumorigenic process could be initiated in the cells of any tissue capable of producing mitochondrial stress signaling following repetitive sub-lethal respiratory damage over prolonged periods. The accumulation of mitochondrial damage over time is what ultimately leads to malignant tumor formation. Acquired abnormalities in mitochondrial function would produce a type of vicious cycle where impaired mitochondrial energy production initiates genome instability and mutability, which then accelerates mitochondrial dysfunction and energy impairment and so on in a cumulative way. An increased dependency on substrate level phosphorylation for survival would follow each round of metabolic and genetic damage thus initiating uncontrolled cell growth and eventual formation of a malignant neoplasm. In other words, the well-documented tumor-associated abnormalities in oncogenes, tumor suppressor genes, and chromosomal imbalances can arise as a consequence of the progressive impairment of mitochondrial function.

### Mitochondrial dysfunction following viral infection

Viruses have long been recognized as the cause of some cancers [152]. It is interesting that several cancer-associated viruses localize to, or accumulate in, the mitochondria. Viral alteration of mitochondrial function could potentially disrupt energy metabolism thus altering expression of tumor suppressor genes and oncogenes over time. Viruses that can affect mitochondrial function include the Rous sarcoma virus, Epstein-Barr virus (EBV), Kaposi's sarcoma-associated herpes virus (KSHV), human papilloma virus (HPV), hepatitis B virus (HBV), hepatitis C virus (HCV), and human T-cell leukemia virus type 1 (HTLV-1) [64,153-155]. Although viral disruption of mitochondrial function will kill most cells through apoptosis following an acute infection, those infected cells that can up-regulate substrate level phosphorylation will survive and potentially produce a neoplasm following chronic infection. Indeed, the hepatitis B × protein (HBx) blocks HIF-1α ubiquitination thus increasing HIF-1α stability and activity in a hypoxia-independent manner [135]. Alterations in calcium homeostasis, ROS production, and expression of NF-kB and HIF-1α are also expected to alter the metabolic state as was previously found for some viral infections [153,154]. It is interesting in this regard that carcinogenesis, whether arising from viral infection or from chemical agent, produces similar impairment in respiratory enzyme activity and mitochondrial function [90]. Thus, viruses can potentially cause cancer through displacement of respiration with substrate level phosphorylation in the infected cells. Alterations in expression of tumor suppressor genes and oncogenes will follow this energy transformation according to the general hypothesis presented here.

### Mitochondrial suppression of tumorigenicity

While the mutator phenotype of cancer can be linked to impaired mitochondrial function, normal mitochondrial function can also suppress tumorigenesis. It is well documented that tumorigenicity can be suppressed when cytoplasm from enucleated normal cells is fused with tumor cells to form cybrids, suggesting that normal mitochondria can suppress the tumorigenic phenotype [156-158]. Singh and co-workers provided additional evidence for the role of mitochondria in the suppression of tumorigenicity by showing that exogenous transfer of wild type mitochondria to cells with depleted mitochondria (rho^0 ^cells) could reverse the altered expression of the APE1 multifunctional protein and the tumorigenic phenotype [113]. On the other hand, introduction of mitochondrial mutations can reverse the anti-tumorigenic effect of normal mitochondria in cybrids [159]. It is also well documented that nuclei from cancer cells can be reprogrammed to form normal tissues when transplanted into normal cytoplasm despite the continued presence of the tumor-associated genomic defects in the cells of the derived tissues [160-162]. These findings indicate that nuclear gene mutations alone cannot account for the origin of cancer and further highlight the dynamic role of mitochondria in the epigenetic regulation of carcinogenesis.

It is expected that the presence of normal mitochondria in tumor cells would restore the cellular redox status, turn off the RTG response, and reduce or eliminate the need for glycolysis (Warburg effect) and glutaminolysis to maintain viability. In other words, normal mitochondrial function would facilitate expression of the differentiated state thereby suppressing the tumorigenic or undifferentiated state. This concept can link mitochondrial function to the long-standing controversy on cellular differentiation and tumorigenicity [5,163]. Respiration is required for the emergence and maintenance of differentiation, while loss of respiration leads to glycolysis, dedifferentiation, and unbridled proliferation [8,25]. These observations are consistent with the general hypothesis presented here, that prolonged impairment of mitochondrial energy metabolism underlies carcinogenesis. New studies are necessary to assess the degree to which cellular energy balance is restored in cybrids and in reprogrammed tumor cells.

### Linking the acquired capabilities of cancer to impaired energy metabolism

Although the mutator phenotype was considered the essential enabling characteristic for manifesting the six hallmarks of cancer, the pathways by which the acquired capabilities of cancer are linked specifically to impaired energy metabolism remain poorly defined. Kromer and Pouyssegur recently provided an overview on how the hallmarks of cancer could be linked to signaling cascades and to the metabolic reprogramming of cancer cells [164]. As the acquired capabilities of self-sufficiency in growth signals, insensitivity to growth inhibitory (antigrowth) signals, and limitless replicative potential are similar, these capabilities can be grouped and discussed together. The acquired capabilities of evasion of programmed cell death, angiogenesis, and metastasis can be discussed separately.

### Growth signaling abnormalities and limitless replicative potential

A central concept in linking abnormalities of growth signaling and replicative potential to impaired energy metabolism is in recognizing that proliferation rather than quiescence is the default state of both microorganisms and metazoans [5,8,165,166]. The cellular default state is the condition under which cells are found when they are freed from any active control. Respiring cells in mature organ systems are quiescent largely because their replicative potential is under negative control through the action of tumor suppressor genes like p53 and the retinoblastoma protein, pRB [144,165]. As p53 function is linked to cellular respiration, prolonged damage to respiration will gradually reduce p53 function thus inactivating the negative control of p53 and of other tumor suppressor genes on cell proliferation.

A persistent impairment in respiratory function will trigger the RTG response, which is necessary for up-regulating the pathways of glycolysis and glutaminolysis in order to maintain the ΔG'_**ATP **_for viability. The RTG response will activate MYC, Ras, HIF-1α, Akt, and m-Tor etc, which are required to facilitate and to sustain up-regulation of substrate level phosphorylation [61,110,113,167,168]. In addition to facilitating the uptake and metabolism of alternative energy substrates through substrate level phosphorylation, MYC and Ras further stimulate cell proliferation [136,169,170]. Part of this mechanism also includes inactivation of pRB, the function of which is dependent on mitochondrial activities and the cellular redox state [144]. Disruption of the pRB signaling pathway will contribute to cell proliferation and neoplasia [6]. Hence, the growth signaling abnormalities and limitless replicative potential of tumor cells can be linked directly to the requirements of glycolysis and glutaminolysis and ultimately to impaired respiration.

It is interesting that RTG signaling also underlies replicative life span extension in budding yeast. Yeast longevity is manifested by the number of buds that a mother cell produces before it dies [110]. The greater the loss of mitochondrial function, the greater is the induction of the RTG response, and the greater the longevity (bud production) [108]. As mitochondrial function declines with age, substrate level phosphorylation becomes necessary to compensate for the lost energy from respiration if a cell is to remain alive. A greater reliance on substrate level phosphorylation will induce oncogene expression and unbridled proliferation, which could underlie in part the enhanced longevity in yeast [110,112,119]. When this process occurs in mammalian cells, however, the phenomenon is referred to as neoplasia or "new growth". We propose that replicative life span extension in yeast and limitless replicative potential in tumor cells can be linked through common bioenergetic mechanisms involving impaired mitochondrial function.

### Linking telomerase to mitochondrial function

Emerging evidence indicates that telomerase, a ribonucleoprotein complex, plays a role in tumor progression [171]. Although still somewhat sparse, data suggest that mitochondrial dysfunction could underlie the relocation of telomerase from the mitochondria, where it seems to have a protective role, to the nucleus where it maintains telomere integrity necessary for limitless replicative potential [172-174]. Interestingly, telomerase activity is high during early embryonic development when anaerobic glycolysis and cell proliferation is high, but telomerase expression is suppressed in adult tissues, where cellular energy is derived largely from respiration. Further studies will be necessary to determine how changes in telomerase expression and subcellular localization could be related to mitochondrial dysfunction, elevated substrate level phosphorylation, and to the limitless replication of tumor cells.

### Evasion of programmed cell death (apoptosis)

Apoptosis is a coordinated process that initiates cell death following a variety of cellular insults. Damage to mitochondrial energy production is one type of insult that can trigger the apoptotic cascade, which ultimately involves release of mitochondrial cytochrome c, activation of intracellular caspases, and death [6]. In contrast to normal cells, acquired resistance to apoptosis is a hallmark of most types of cancer cells [6]. The evasion of apoptosis is a predictable physiological response of tumor cells that up-regulate substrate level phosphorylation for energy production following respiratory damage during the protracted process of carcinogenesis. Only those cells capable of making the gradual energy transition from respiration to substrate level phosphorylation in response to respiratory damage will be able to evade apoptosis. Cells unable to make this energy transition will die and thus never become tumor cells.

Numerous findings indicate that the genes and signaling pathways needed to up-regulate and sustain substrate level phosphorylation are themselves anti-apoptotic. For example, sustained glycolysis requires participation of mTOR, MYC, Ras, HIF-1α, and the IGF-1/PI3K/Akt signaling pathways [28,110,112,113,128,168]. The up-regulation of these genes and pathways together with inactivation of tumor suppressor genes like p53, which is required to initiate apoptosis, will disengage the apoptotic-signaling cascade thus preventing programmed cell death [142].

Abnormalities in the mitochondrial membrane potential (ΔΨ_m_) can also induce expression of known anti-apoptotic genes (Bcl2 and Ccl-X_L_) [111]. Tumor cells will continue to evade apoptosis as long as they have access to glucose and glutamine, which are required to maintain substrate level phosphorylation. Glycolytic tumor cells, however, can readily express a robust apoptotic phenotype if their glucose supply is targeted. This was clearly illustrated in experimental brain tumors using calorie restriction [168,175,176]. Hence, the evasion of apoptosis in tumor cells can be linked directly to a dependency on substrate level phosphorylation, which itself is a consequence of impaired respiratory function.

### Sustained vascularity (angiogenesis)

Angiogenesis involves neovascularization or the formation of new capillaries from existing blood vessels and is associated with the processes of tissue inflammation, wound healing, and tumorigenesis [123,124,177,178]. Angiogenesis is required for most tumors to grow beyond an approximate size of 0.2-2.0 mm [179]. Vascularity is necessary in order to provide the tumor with essential energy nutrients to include glucose and glutamine, and to remove toxic tumor waste products such as lactic acid and ammonia [49]. In addition to its role in up-regulating glycolysis in response to hypoxia, HIF-1α is also the main transcription factor for vascular endothelial growth factor (VEGF), which stimulates angiogenesis [168,180-182]. HIF-1α is part of the IGF-1/PI3K/Akt signaling pathway that also indirectly influences expression of β FGF, another key angiogenesis growth factor [168,183]. Hence the sustained vascularity of tumors can be linked mechanistically to the metabolic requirements of substrate level phosphorylation necessary for tumor cell survival.

### Invasion and metastasis

Metastasis is the general term used to describe the spread of cancer cells from the primary tumor to surrounding tissues and to distant organs and is a primary cause of cancer morbidity and mortality [6,184,185]. Metastasis involves a complex series of sequential and interrelated steps. In order to complete the metastatic cascade, cancer cells must detach from the primary tumor, intravasate into the circulation and lymphatic system, evade immune attack, extravasate at a distant capillary bed, and invade and proliferate in distant organs [185-189]. Metastatic cells also establish a microenvironment that facilitates angiogenesis and proliferation, resulting in macroscopic, malignant secondary tumors. A difficulty in better characterizing the molecular mechanisms of metastasis comes in large part from the lack of animal models that manifest all steps of the cascade. Tumor cells that are naturally metastatic should not require intravenous injection in animal models to initiate the metastatic phenotype [190,191]. *In vitro *models, on the other hand, do not replicate all the steps required for systemic metastasis *in vivo*. Although the major steps of metastasis are well documented, the process by which metastatic cells arise from within populations of non-metastatic cells of the primary tumor is largely unknown [185,192,193].

Several mechanisms have been advanced to account for the origin of metastasis. The epithelial-mesenchymal transition (EMT) posits that metastatic cells arise from epithelial cells through a step-wise accumulation of gene mutations that eventually transform an epithelial cell into a tumor cell with mesenchymal features [6,100,194-196]. The idea comes from findings that many cancers generally arise in epithelial tissues where abnormalities in cell-cell and cell-matrix interactions occur during tumor progression. Eventually neoplastic cells emerge that appear as mesenchymal cells, which lack cell-cell adhesion and are dysmorphic in shape [185]. These transformed epithelial cells eventually acquire the multiple effector mechanisms of metastasis [185]. Recent studies suggest that ectopic co-expression of only two genes might be all that is necessary to facilitate EMT in some gliomas [197]. Considerable controversy surrounds the EMT hypothesis of metastasis, however, as EMT is not often detected in tumor pathological preparations [198,199].

The macrophage hypothesis of metastasis suggests that metastatic cells arise following fusions of macrophages or bone marrow derived hematopoietic cells with committed tumor cells [193,200,201]. It is well documented that metastatic cancer cells, arising from a variety of tissues, possess numerous properties of macrophages or cells of myeloid lineage including phagocytosis and fusogenicity [190,202-208]. Macrophages and other types of myeloid cells are already genetically programmed to enter and exit tissues. Many of the normal behaviors of macrophages elaborate each step of the metastatic cascade [204]. Fusion of a myeloid cell (macrophage) with a tumor cell could produce a hybrid cell possessing the replicative capacity of the tumor cell and the properties of macrophages including the invasive and inflammatory properties [193,205,209]. As myeloid cells are also part of the immune system, evasion of immune surveillance would be another expected characteristic of metastatic cells derived from macrophage-like cells [205]. Indeed, metastatic melanoma cells can phagocytose live T-cells, which are supposed to kill the tumor cells [210].

Fusions among metastatic myeloid cells at the primary tumor site could, through reprogramming strategies, also produce functional epithelial cells at secondary sites potentially simulating the histological characteristics of the original tissue of origin [200,211,212]. The macrophage fusion hypothesis would also fit with the roles of hematopoietic stem cells in the metastatic niche [208,213]. While the fusion hypothesis is attractive, it would be an exception to the observations showing suppressed tumorigenicity following hybridization between normal cells and tumor cells [163], though some exceptions have been reported [205,206]. However, neither the EMT hypothesis nor the macrophage fusion hypothesis link the origin of metastasis to the Warburg effect or to impaired energy metabolism.

Recent findings of cardiolipin abnormalities in systemic metastatic mouse tumor cells with macrophage properties can link metastasis to impaired respiratory function in these cells [73,190,204]. Most tissues contain resident phagocytes as part of their histoarchitecture or stroma [214]. Tumor associated macrophages (TAM) also become a major cell type in many cancers [215]. While TAM can facilitate the invasive and metastatic properties of tumor cells [213,216], metastatic tumor cells can also express several properties of TAM [190,204].

Damage to the respiratory capacity of resident tissue phagocytes, TAM, or macrophage hybrids would trigger a RTG response, force a reliance on substrate level phosphorylation for energy, and eventually, over time, lead to dysregulated growth and genomic instability as described in the general hypothesis. Metastatic behavior would be an expected outcome following impaired respiratory function in hematopoietic or myeloid-type cells, as macrophages are already mesenchymal cells that embody the capacity to degrade the extracellular matrix, to enter and to exit tissues from the blood stream, to migrate through tissues, and to survive in hypoxic environments. A sampling of human metastatic cancers with properties of macrophage-like cells include brain [204,217-220], breast [221-225], lung [202,225-229], skin [203,205,209,210,230-233], gastric [234], colon [235,236], pancreas [237,238], bladder [239], kidney [240], ovarian [241,242], and muscle [243,244]. It is important to mention that these macrophage properties are expressed in the tumor cells themselves and are not to be confused with similar properties expressed in the non-neoplastic TAM, which are also present in tumors and can facilitate tumor progression [190,213,215,216,245]. Poor prognosis is generally associated with those cancers that display characteristics of macrophages [210,221]. Hence, damage to the respiratory capacity of myeloid or macrophage-like cells would produce "rogue macrophages" leading to cancers with the highest metastatic behavior.

The plethora of the cell surface molecules thought to participate in metastatic tumor cell behavior are also expressed on myeloid cells especially macrophages [185,213]. A robust Warburg effect in human metastatic lesions, detected with combined ^18^F-fluorodeoxyglucose-positron emission tomography imaging, indicates that metastatic cells have impaired energy metabolism like that of most cancer cells [18,20,246]. Hence, invasion and metastasis can be linked to impaired energy metabolism if this impairment occurs in cells of hematopoietic or myeloid origin.

### Connecting the links

**Figure 2:**
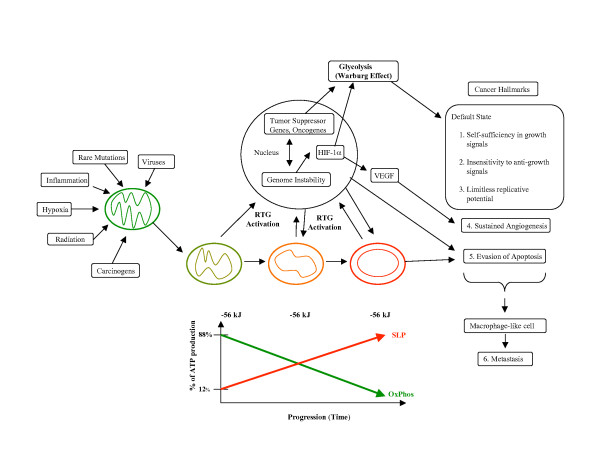
**Linking the hallmarks of cancer to impaired energy metabolism**. See text for discussion. SLP and OxPhos represent substrate level phosphorylation and oxidative phosphorylation, respectively. The progressive damage to mitochondria during carcinogenesis is illustrated with a change in shape.

The path from normal cell physiology to malignant behavior, where all major cancer hallmarks are expressed, is depicted in Figure 2 and is based on the evidence reviewed above. Any unspecific condition that damages a cell's oxidative phosphorylation, but is not severe enough to induce apoptosis, can potentially initiate the path to a malignant cancer. Some of the many unspecific conditions contributing to carcinogenesis can include inflammation, carcinogens, radiation (ionizing or ultraviolet), intermittent hypoxia, rare germline mutations, viral infections, and disruption of tissue morphogenetic fields. Any of these conditions can damage the structure and function of mitochondria thus activating a specific RTG response in the damaged cell. If the mitochondrial damage persists, the RTG response will persist. Uncorrected mitochondrial damage will require a continuous compensatory energy response involving substrate level phosphorylation in order to maintain the ΔG'_**ATP **_of approximately -56 kJ/mol for cell viability. Tumor progression is linked to a greater dependence on substrate level phosphorylation, which eventually becomes irreversible. As the integrity of the nuclear genome is dependent on the efficiency of mitochondrial energy production, the continued impairment of mitochondrial energy production will gradually undermine nuclear genome integrity leading to a mutator phenotype and a plethora of somatic mutations. Activation of oncogenes, inactivation of tumor suppressor genes, and aneuploidy will be the consequence of protracted mitochondrial dysfunction. These gene abnormalities will contribute further to mitochondrial dysfunction while also enhancing those energy pathways needed to up-regulate and sustain substrate level phosphorylation. The greater the dependency on substrate level phosphorylation over time the greater will be the degree of malignancy. Damage to the respiratory capacity of tissue myeloid cells can also produce invasive and metastatic properties according to the macrophage hypothesis of metastasis. This metabolic scenario can account for all major acquired characteristics of cancer to include the Warburg effect.

### Implications of the hypothesis to cancer management

If cancer is primarily a disease of energy metabolism as reviewed here, then rational approaches to cancer management can be found in therapies that specifically target energy metabolism. Although mitochondrial replacement therapy could in principle restore a more normal energy metabolism and differentiated state to tumor cells, it is unlikely that this therapeutic approach would be available in the foreseeable future. However, numerous studies show that dietary energy restriction is a general metabolic therapy that naturally lowers circulating glucose levels and significantly reduces growth and progression of numerous tumor types to include cancers of the mammary, brain, colon, pancreas, lung, and prostate [10,247-256]. The influence of energy restriction on tumor growth, however, can depend on host background and tumor growth site, as energy restriction is effective in reducing the U87 human glioma when grown orthotopically in the brain of immunodeficient SCID mice [175], but not when grown outside the brain in non-obese diabetic SCID mice [257]. Nevertheless, the bulk of evidence indicates that dietary energy restriction can retard the growth rate of many tumors regardless of the specific genetic defects expressed within the tumor.

#### Targeting Glucose

Reduced glucose availability will target aerobic glycolysis and the pentose phosphate shunt; pathways required for the survival and proliferation of many types of tumor cells. Dietary energy restriction specifically targets the IGF-1/PI3K/Akt/HIF-1α signaling pathway, which underlies several cancer hallmarks to include cell proliferation, evasion of apoptosis, and angiogenesis [168,175,176,250,251,254,258-265]. Calorie restriction also causes a simultaneous down-regulation of multiple genes and metabolic pathways regulating glycolysis [266-268]. This is important, as enhanced glycolysis is required for the rapid growth and survival of many tumor cells [21,22]. In addition, recent findings suggest that a large subset of gliomas have acquired mutations in the TCA cycle gene, isocitrate dehydrogenase (*IDH1) *[105]. Such mutations are expected to limit the function of the TCA cycle, thus increasing the glycolytic dependence of these tumors. Tumors with these types of mutations could be especially vulnerable to management through dietary energy restriction. Hence, dietary energy or calorie restriction can be considered a broad-spectrum, non-toxic metabolic therapy that inhibits multiple signaling pathways required for progression of malignant tumors regardless of tissue origin.

Besides lowering circulating glucose levels, dietary energy restriction elevates circulating levels of fatty acids and ketone bodies (β-hydroxybutyrate and acetoacetate) [266,269,270]. Fats and especially ketone bodies can replace glucose as a primary metabolic fuel under calorie restriction. This is a conserved physiological adaptation that evolved to spare protein during periods of starvation [271,272]. Many tumors, however, have abnormalities in the genes and enzymes needed to metabolize ketone bodies for energy [273-275]. A transition from carbohydrate to ketones for energy is a simple way to target energy metabolism in glycolysis-dependent tumor cells while enhancing the metabolic efficiency of normal cells [276,277]. The shift from the metabolism of glucose to the metabolism of ketone bodies for energy is due largely to the shift in circulating levels of insulin and glucagon, key hormones that mediate energy metabolism. Insulin, which stimulates glycolysis, is reduced under dietary restriction, while glucagon, which inhibits glycolysis and mobilizes fats, is increased. Glucose reduction not only reduces insulin, but also reduces circulating levels of IGF-1, which is necessary for driving tumor cell metabolism and growth [168,278]. Glucocorticoids, which enhance glucagon action and the stress response, are also elevated under dietary energy restriction [261]. The shift in levels of these metabolic hormones would place greater physiological stress on the tumor cells than on normal cells since the tumor cells lack metabolic flexibility due to accumulated genetic mutations [10,15,277].

Inferences that tumor cells have a growth advantage over normal cells are inconsistent with principles of evolutionary biology [10,277]. Although viewed as a growth advantage, the dysregulated growth of tumor cells is actually an aberrant phenotype. How can tumor cells that express multiple mutations and mitochondrial abnormalities be more "fit" or "advantaged" than normal cells that possess a flexible genome, normal respiratory capacity, and adaptive versatility? The short answer is that they are not. Normal cells can grow much faster than tumor cells during normal wound repair. Metabolism of ketone bodies and fatty acids for energy requires inner mitochondrial membrane integrity and efficient respiration, which tumor cells largely lack [10,273,278]. In contrast to the tumor cells, normal cells evolved to survive extreme shifts in the physiological environment and can readily adapt to fat metabolism when glucose becomes limiting. Glucose transporter expression is higher in mouse brain tumor cells than in neighboring normal cells when circulating glucose levels are high, but the transporter phenotype of these cells becomes reversed under dietary energy restriction [168]. These findings highlight the different responses to energy stress between the metabolically incompetent tumor cells and competent normal cells. Consequently, a shift in energy metabolism from glucose to ketone bodies protects respiratory competent normal cells while targeting the genetically defective and respiratory challenged tumor cells, which depend more heavily on glycolysis than normal cells for survival [10,278,279].

Proof of concept for cancer metabolic therapy was illustrated for the management of malignant astrocytoma in mice, and malignant glioma in children [273,276,280]. Prostate and gastric cancer also appears manageable using low carbohydrate ketogenic diets [252,281,282]. Recent studies show that dietary energy restriction enhances phosphorylation of adenosine monophosphate kinase (AMPK), which induces apoptosis in glycolytic-dependent astrocytoma cells, but protects normal brain cells from death [283]. This further illustrates the differential response of normal cells and tumor cells to energy stress.

A possible concern is how any therapy, which reduces food intake and body weight, can be recommended to individuals who might be losing body weight because of cancer cachexia. Cancer cachexia generally involves anorexia, weight loss, muscle atrophy, and anemia [284,285]. Although some cancer patients could be obese, rapid weight loss from cachexia involving both proteins and fat is a health concern [285]. It is important to recognize that pro-cachexia molecules such as proteolysis-inducing factor are released from the tumor cells into the circulation and contribute to the cachexia phenotype [286,287]. By targeting the glycolytically active tumor cells that produce pro-cachexia molecules, restricted diet therapies can potentially reduce tumor cachexia [278,287]. These therapies could be supplemented with omega-3 fatty acids, which can also reduce the cachexia phenotype [285]. Omega-3 fatty acids from fish oil also have the benefit of maintaining low glucose while elevating ketone levels. Once the tumor becomes managed, individuals can increase caloric consumption to achieve weight gain.

Metabolic therapies involving calorie restriction should be effective in targeting energy-defective cells within a given tumor, and for managing a broad range of glycolysis-dependent tumors. There are no known drugs that can simultaneously target as many tumor-associated signaling pathways as can calorie restriction [168]. Hence, energy restriction can be a cost-effective adjuvant therapy to traditional chemo- or radiation therapies, which are more toxic, costly, and generally less focused in their therapeutic action, than is dietary energy restriction.

In addition to dietary energy restriction, several small molecules that target aerobic glycolysis are under consideration as novel tumor therapeutics to include 2-deoxyglucose, lonidamine, 3-bromopyruvate, imatinib, oxythiamine, and 6-aminonicotinimide among others [129,288-290]. Toxicity can become an issue, however, as some of these compounds target pathways other than glycolysis or nucleotide synthesis and high dosages are sometimes required to achieve efficacy *in vivo*. A recent study found significant therapeutic synergy in combining low doses of 2-deoxyglucose with a calorie restricted ketogenic diet for managing malignant astrocytoma in mice [291].

It appears that the therapeutic efficacy of anti-glycolytic cancer drugs could be significantly enhanced when combined with dietary energy restriction. The administration of anti-glycolytic drugs together with energy restricted diets, which lower circulating glucose levels while elevating ketone levels, could act as a powerful double "metabolic punch" for the rapid killing of glycolysis dependent tumor cells. This therapeutic approach could open new avenues in cancer drug development, as many drugs that might have minimal therapeutic efficacy or high toxicity when administered alone could become therapeutically relevant and less toxic when combined with energy restricted diets.

#### Targeting the microenvironment

Some tumors behave as wounds that do not heal [292]. Growth factors and cytokines released by fibroblasts and macrophages, cells programmed to heal wounds, can actually provoke chronic inflammation and tumor progression [213,245]. Part of the wound healing process also involves degradation of the extracellular matrix and enhancement of angiogenesis, which further contribute to tumor progression [180,213]. Dietary energy restriction targets inflammation and the signaling pathways involved with driving tumor angiogenesis [168,258,293]. Indeed, calorie restriction is considered a simple and effective therapy for targeting tumor angiogenesis and inflammation [176,250,279]. As calorie or dietary energy restriction is a systemic therapy that simultaneously targets both the tumor cells as well as the tumor microenvironment, this approach can be effective in retarding tumor progression.

#### Targeting Glutamine

Although dietary energy restriction and anti-glycolytic cancer drugs will have therapeutic efficacy against many tumors that depend largely on glycolysis and glucose for growth, these therapeutic approaches could be less effective against those tumor cells that depend more heavily on glutamine than on glucose for energy [47,65-67]. Glutamine is a major energy metabolite for many tumor cells and especially for cells of hematopoietic or myeloid lineage [47,49,294,295]. This is important as cells of myeloid lineage are considered the origin of many metastatic cancers [17,190,204,221,230]. Moreover, glutamine is necessary for the synthesis of those cytokines involved in cancer cachexia including tumor necrosis factor alpha, (TNF-α) and the interleukins 1 and 6 (IL-1 and -6) [66,284,295,296]. This further indicates a metabolic linkage between metastatic cancer and myeloid cells, e.g., macrophages. It therefore becomes important to also consider glutamine targeting for the metabolic management of metastatic cancer.

Glutamine can be deaminated to glutamate and then metabolized to α-ketoglutarate, a key metabolite of the TCA cycle [49,67]. This occurs either through transamination or through enhanced glutamate dehydrogenase activity depending on the availability of glucose [67]. Besides generating energy through substrate level phosphorylation in the TCA cycle, i.e., transphosphorylation of GTP to ATP, the anapleurotic effect of glutamine can also elevate levels of metabolic substrates, which stimulate glycolysis [49,66]. Glutamine metabolism can be targeted in humans using the glutamine binding drug, phenylacetate, or the glutamine analogue DON (6-Diazo-5-oxo-L-norleucine) [297]. Toxicity, however, can be an issue in attempts to target glutamine metabolism using DON [130,294]. Recent studies suggest that the green tea polyphenol (EGCG) could target glutamine metabolism by inhibiting glutamate dehydrogenase activity under low glucose conditions [67]. This and other glutamine-targeting strategies could be even more effective when combined with energy restricting diets, which lower glucose levels while elevating ketone bodies. Hence, effective non-toxic targeting of both glucose and glutamine metabolism should be a simple therapeutic approach for the global management of most localized and metastatic cancers.

### Implications of the hypothesis to cancer prevention

If impaired mitochondrial energy metabolism underlies the origin of most cancers as proposed here, then protecting mitochondria from damage becomes a logical and simple approach for preventing cancer. It is well documented that the incidence of cancer can be significantly reduced by avoiding exposure to those agents or conditions that provoke tissue inflammation such as smoking, alcohol, carcinogenic chemicals, ionizing radiation, obesity etc [1,25,298]. Chronic inflammation, regardless of origin, damages tissue morphogenetic fields that eventually produce neoplastic cells [123,124,166]. Part of this tissue damage will involve injury to the mitochondria in the affected cells. The prevention of inflammation and damage to the tissue microenvironment will go far in reducing the incidence of most cancers. Vaccines against some oncogenic viruses can also reduce the incidence of cancers, as these viruses can damage mitochondria in infected tissues. Hence, simply reducing exposure to cancer risk factors, which produce chronic inflammation and mitochondrial damage, will reduce the incidence of at least 80% of all cancers [1,25]. In principle, there are few chronic diseases more easily preventable than cancer.

In addition to avoiding exposure to established cancer risk factors, the metabolism of ketone bodies protects the mitochondria from inflammation and damaging ROS. ROS production increases naturally with age and damages cellular proteins, lipids, and nucleic acids. Accumulation of ROS decreases the efficiency of mitochondrial energy production. The origin of mitochondrial ROS comes largely from the spontaneous reaction of molecular oxygen (O_2_) with the semiquinone radical of coenzyme Q, ^.^QH, to generate the superoxide radical O_2_-^. ^[40,84,299]. Coenzyme Q is a hydrophobic molecule that resides in the inner mitochondrial membrane and is essential for electron transfer. Ketone body metabolism increases the ratio of the oxidized form to the fully reduced form of coenzyme Q (CoQ/CoQH_2_) [40]. Oxidation of the coenzyme Q couple reduces the amount of the semiquinone radical, thus decreasing superoxide production [84].

Since the cytosolic free NADP^+^/NADPH concentration couple is in near equilibrium with the glutathione couple, ketone body metabolism will also increase the reduced form of glutathione thus facilitating destruction of hydrogen peroxide [10,84,300]. The reduction of free radicals through ketone body metabolism will therefore reduce tissue inflammation provoked by ROS while enhancing the energy efficiency of mitochondria. Ketone bodies are not only a more efficient metabolic fuel than glucose, but also possess anti-inflammatory potential. Metabolism of ketone bodies for energy will maintain mitochondrial health and efficiency thus reducing the incidence of cancer.

The simplest means of initiating the metabolism of ketone bodies is through dietary energy restriction with adequate nutrition. It is important to emphasize adequate nutrition, as calorie restriction associated with malnutrition can potentially increase cancer incidence [301-303]. Consequently, consumption of foods containing the active groups of respiratory enzymes (iron salts, riboflavin, nicotinamide, and pantothenic acid) could be effective in maintaining health when combined with dietary energy restriction [25]. The lowering of circulating glucose levels through calorie restriction facilitates the uptake and metabolism of ketone bodies for use as an alternative respiratory fuel [84,273,278]. The metabolism of ketone bodies increases succinate dehydrogenase activity while enhancing the overall efficiency of energy production through respiration [304,305]. In essence, dietary energy restriction and ketone body metabolism delays entropy [270]. As cancer is a disease of accelerated entropy [8,25], dietary energy restriction targets the very essence of the disease.

It is well documented that dietary energy restriction can reduce the incidence of both inherited and acquired cancers in experimental animals [256,258,306-309]. Evidence also indicates that dietary energy restriction can reduce the incidence of several human cancers [310,311]. The implementation of periodic dietary energy restriction, which targets multiple cancer provoking factors, can be a simple and cost effective life-style change that is capable of reducing the incidence of cancer. Dietary energy restriction in rodents, however, is comparable to water only therapeutic fasting or to very low caloric diets (500-600 kcal/day) in humans [270]. In light of this fact, it remains to be determined if members of our species are willing or motivated enough to adopt the life style changes necessary to prevent cancer.

## Conclusions

Evidence is reviewed supporting a general hypothesis that cancer is primarily a disease of energy metabolism. All of the major hallmarks of the disease can be linked to impaired mitochondrial function. In order to maintain viability, tumor cells gradually transition to substrate level phosphorylation using glucose and glutamine as energy substrates. While cancer causing germline mutations are rare, the abundance of somatic genomic abnormalities found in the majority of cancers can arise as a secondary consequence of mitochondrial dysfunction. Once established, somatic genomic instability can contribute to further mitochondrial defects and to the metabolic inflexibility of the tumor cells. Systemic metastasis is the predicted outcome following protracted mitochondrial damage to cells of myeloid origin. Tumor cells of myeloid origin would naturally embody the capacity to exit and enter tissues. Two major conclusions emerge from the hypothesis; first that many cancers can regress if energy intake is restricted and, second, that many cancers can be prevented if energy intake is restricted. Consequently, energy restricted diets combined with drugs targeting glucose and glutamine can provide a rational strategy for the longer-term management and prevention of most cancers.

## Competing interests

The authors declare that they have no competing interests.

## Authors' contributions

TNS wrote the manuscript. LMS contributed to the general outline of topic presentation, editorial assistance, and to discussion of key issues. Both authors read and approved the final manuscript.

## References

[B1] Anand P Kunnumakkara AB Sundaram C Harikumar KB Tharakan ST Lai OS Sung B Aggarwal BB Cancer is a preventable disease that requires major lifestyle changes Pharm Res 2008 25 2097 2116 10.1007/s11095-008-9661-9 18626751 PMC2515569

[B2] Bailar JC Gornik HL Cancer undefeated N Engl J Med 1997 336 1569 1574 10.1056/NEJM199705293362206 9164814

[B3] Sonnenschein C Soto AM Theories of carcinogenesis: an emerging perspective Semin Cancer Biol 2008 18 372 377 10.1016/j.semcancer.2008.03.012 18472276 PMC2730644

[B4] Baker SG Kramer BS Paradoxes in carcinogenesis: new opportunities for research directions BMC Cancer 2007 7 151 10.1186/1471-2407-7-151 17683619 PMC1993836

[B5] Soto AM Sonnenschein C The somatic mutation theory of cancer: growing problems with the paradigm? Bioessays 2004 26 1097 1107 10.1002/bies.20087 15382143

[B6] Hanahan D Weinberg RA The hallmarks of cancer Cell 2000 100 57 70 10.1016/S0092-8674(00)81683-9 10647931

[B7] Loeb LA A mutator phenotype in cancer Cancer Res 2001 61 3230 3239 11309271

[B8] Szent-Gyorgyi A The living state and cancer Proc Natl Acad Sci USA 1977 74 2844 2847 10.1073/pnas.74.7.2844 268635 PMC431314

[B9] Roth DB Gellert M New guardians of the genome Nature 2000 404 823 825 10.1038/35009180 10786775

[B10] Seyfried TN Mukherjee P Targeting energy metabolism in brain cancer: review and hypothesis Nutr Metab (Lond) 2005 2 30 10.1186/1743-7075-2-30 16242042 PMC1276814

[B11] Semenza GL Artemov D Bedi A Bhujwalla Z Chiles K Feldser D Laughner E Ravi R Simons J Taghavi P Zhong H 'The metabolism of tumours': 70 years later Novartis Found Symp 2001 240 251 260 discussion 260-254full_text 11727934

[B12] Ristow M Oxidative metabolism in cancer growth Curr Opin Clin Nutr Metab Care 2006 9 339 345 10.1097/01.mco.0000232892.43921.98 16778561

[B13] Gatenby RA Gillies RJ Why do cancers have high aerobic glycolysis? Nat Rev Cancer 2004 4 891 899 10.1038/nrc1478 15516961

[B14] Gogvadze V Orrenius S Zhivotovsky B Mitochondria in cancer cells: what is so special about them? Trends Cell Biol 2008 18 165 173 10.1016/j.tcb.2008.01.006 18296052

[B15] Lengauer C Kinzler KW Vogelstein B Genetic instabilities in human cancers Nature 1998 396 643 649 10.1038/25292 9872311

[B16] Wokolorczyk D Gliniewicz B Sikorski A Zlowocka E Masojc B Debniak T Matyjasik J Mierzejewski M Medrek K Oszutowska D Suchy J Gronwald J Teodorczyk U Huzarski T Byrski T Jakubowska A Gorski B Wetering T van de Walczak S Narod SA Lubinski J Cybulski C A range of cancers is associated with the rs6983267 marker on chromosome 8 Cancer Res 2008 68 9982 9986 10.1158/0008-5472.CAN-08-1838 19047180

[B17] Nowell PC Tumor progression: a brief historical perspective Semin Cancer Biol 2002 12 261 266 10.1016/S1044-579X(02)00012-3 12147207

[B18] Frezza C Gottlieb E Mitochondria in cancer: Not just innocent bystanders Semin Cancer Biol 2008 19101633 10.1016/j.semcancer.2008.11.008

[B19] Gatenby RA Gillies RJ Glycolysis in cancer: a potential target for therapy Int J Biochem Cell Biol 2007 39 1358 1366 10.1016/j.biocel.2007.03.021 17499003

[B20] Heiden MG Vander Cantley LC Thompson CB Understanding the Warburg effect: the metabolic requirements of cell proliferation Science 2009 324 1029 1033 10.1126/science.1160809 19460998 PMC2849637

[B21] Ortega AD Sanchez-Arago M Giner-Sanchez D Sanchez-Cenizo L Willers I Cuezva JM Glucose avidity of carcinomas Cancer Lett 2009 276 125 135 10.1016/j.canlet.2008.08.007 18790562

[B22] Altenberg B Greulich KO Genes of glycolysis are ubiquitously overexpressed in 24 cancer classes Genomics 2004 84 1014 1020 10.1016/j.ygeno.2004.08.010 15533718

[B23] Warburg O Richard R Smith The Metabolism of Tumours New York 1931

[B24] Warburg O On the origin of cancer cells Science 1956 123 309 314 10.1126/science.123.3191.309 13298683

[B25] Warburg O The prime cause of cancer and prevention - Part 2 Annual meeting of Nobelists at Lindau, Germany 1969 http://www.hopeforcancer.com/OxyPlus.htm

[B26] Moreno-Sanchez R Rodriguez-Enriquez S Saavedra E Marin-Hernandez A Gallardo-Perez JC The bioenergetics of cancer: is glycolysis the main ATP supplier in all tumor cells? Biofactors 2009 35 209 225 10.1002/biof.31 19449450

[B27] Bonnet S Archer SL Allalunis-Turner J Haromy A Beaulieu C Thompson R Lee CT Lopaschuk GD Puttagunta L Bonnet S Harry G Hashimoto K Porter CJ Andrade MA Thebaud B Michelakis ED A mitochondria-K+ channel axis is suppressed in cancer and its normalization promotes apoptosis and inhibits cancer growth Cancer Cell 2007 11 37 51 10.1016/j.ccr.2006.10.020 17222789

[B28] Semenza GL HIF-1 mediates the Warburg effect in clear cell renal carcinoma J Bioenerg Biomembr 2007 39 231 234 10.1007/s10863-007-9081-2 17551816

[B29] Moreno-Sanchez R Rodriguez-Enriquez S Marin-Hernandez A Saavedra E Energy metabolism in tumor cells Febs J 2007 274 1393 1418 10.1111/j.1742-4658.2007.05686.x 17302740

[B30] Aisenberg AC The Glycolysis and Respiration of Tumors 1961 New York, Academic Press

[B31] Fantin VR Leder P Mitochondriotoxic compounds for cancer therapy Oncogene 2006 25 4787 4797 10.1038/sj.onc.1209599 16892091

[B32] Hervouet E Demont J Pecina P Vojtiskova A Houstek J Simonnet H Godinot C A new role for the von Hippel-Lindau tumor suppressor protein: stimulation of mitochondrial oxidative phosphorylation complex biogenesis Carcinogenesis 2005 26 531 539 10.1093/carcin/bgi001 15604095

[B33] Weinhouse S On respiratory impairment in cancer cells Science 1956 124 267 269 10.1126/science.124.3215.267 13351638

[B34] Weinhouse S The Warburg hypothesis fifty years later Z Krebsforsch Klin Onkol Cancer Res Clin Oncol 1976 87 115 126 10.1007/BF00284370 136820

[B35] Krebs H Otto Warburg: Cell Physiologist, Biochemist, and Eccentric 1981 Oxford, Clarendon

[B36] Kim JW Dang CV Cancer's molecular sweet tooth and the Warburg effect Cancer Res 2006 66 8927 8930 10.1158/0008-5472.CAN-06-1501 16982728

[B37] Hsu PP Sabatini DM Cancer cell metabolism: Warburg and beyond Cell 2008 134 703 707 10.1016/j.cell.2008.08.021 18775299

[B38] Shaw RJ Glucose metabolism and cancer Current opinion in cell biology 2006 18 598 608 10.1016/j.ceb.2006.10.005 17046224

[B39] Jones RG Thompson CB Tumor suppressors and cell metabolism: a recipe for cancer growth Genes Dev 2009 23 537 548 10.1101/gad.1756509 19270154 PMC2763495

[B40] Veech RL Chance B Kashiwaya Y Lardy HA Cahill GF Jr Ketone bodies, potential therapeutic uses IUBMB Life 2001 51 241 247 10.1080/152165401753311780 11569918

[B41] Kocherginsky N Acidic lipids, H(+)-ATPases, and mechanism of oxidative phosphorylation Physico-chemical ideas 2009 99 20 41 10.1016/j.pbiomolbio.2008.10.013 19049812

[B42] Veech RL Kashiwaya Y Gates DN King MT Clarke K The energetics of ion distribution: the origin of the resting electric potential of cells IUBMB Life 2002 54 241 252 10.1080/15216540215678 12587974

[B43] Veech RL Lawson JW Cornell NW Krebs HA Cytosolic phosphorylation potential J Biol Chem 1979 254 6538 6547 36399

[B44] Donnelly M Scheffler IE Energy metabolism in respiration-deficient and wild type Chinese hamster fibroblasts in culture J Cell Physiol 1976 89 39 51 10.1002/jcp.1040890105 8468

[B45] Baggetto LG Deviant energetic metabolism of glycolytic cancer cells Biochimie 1992 74 959 974 10.1016/0300-9084(92)90016-8 1477140

[B46] Weinberg JM Venkatachalam MA Roeser NF Nissim I Mitochondrial dysfunction during hypoxia/reoxygenation and its correction by anaerobic metabolism of citric acid cycle intermediates Proc Natl Acad Sci USA 2000 97 2826 2831 10.1073/pnas.97.6.2826 10717001 PMC16014

[B47] Reitzer LJ Wice BM Kennell D Evidence that glutamine, not sugar, is the major energy source for cultured HeLa cells J Biol Chem 1979 254 2669 2676 429309

[B48] Schwimmer C Lefebvre-Legendre L Rak M Devin A Slonimski PP di Rago JP Rigoulet M Increasing mitochondrial substrate-level phosphorylation can rescue respiratory growth of an ATP synthase-deficient yeast J Biol Chem 2005 280 30751 30759 10.1074/jbc.M501831200 15975925

[B49] DeBerardinis RJ Is cancer a disease of abnormal cellular metabolism?. New angles on an old idea Genet Med 2008 10 767 777 10.1097/GIM.0b013e31818b0d9b 18941420 PMC2782690

[B50] Phillips D Aponte AM French SA Chess DJ Balaban RS Succinyl-CoA synthetase is a phosphate target for the activation of mitochondrial metabolism Biochemistry 2009 48 7140 7149 10.1021/bi900725c 19527071 PMC2766921

[B51] Wallace DC Mitochondria and cancer: Warburg addressed Cold Spring Harb Symp Quant Biol 2005 70 363 374 10.1101/sqb.2005.70.035 16869773

[B52] Pedersen PL Tumor mitochondria and the bioenergetics of cancer cells Prog Exp Tumor Res 1978 22 190 274 149996 10.1159/000401202

[B53] Wu M Neilson A Swift AL Moran R Tamagnine J Parslow D Armistead S Lemire K Orrell J Teich J Chomicz S Ferrick DA Multiparameter metabolic analysis reveals a close link between attenuated mitochondrial bioenergetic function and enhanced glycolysis dependency in human tumor cells Am J Physiol Cell Physiol 2007 292 C125 136 10.1152/ajpcell.00247.2006 16971499

[B54] Fantin VR St-Pierre J Leder P Attenuation of LDH-A expression uncovers a link between glycolysis, mitochondrial physiology, and tumor maintenance Cancer Cell 2006 9 425 434 10.1016/j.ccr.2006.04.023 16766262

[B55] Colowick SP The status of Warburg's theory of glycolysis and respiration in tumors Quart Rev Biol 1961 36 273 276 10.1086/403479

[B56] Zu XL Guppy M Cancer metabolism: facts, fantasy, and fiction Biochem Biophys Res Commun 2004 313 459 465 10.1016/j.bbrc.2003.11.136 14697210

[B57] Burk D Schade AL On respiratory impairment in cancer cells Science 1956 124 270 272 13351640

[B58] Chance B Hess B Spectroscopic evidence of metabolic control Science 1959 129 700 708 10.1126/science.129.3350.700 13635004

[B59] Samudio I Fiegl M Andreeff M Mitochondrial uncoupling and the Warburg effect: molecular basis for the reprogramming of cancer cell metabolism Cancer Res 2009 69 2163 2166 10.1158/0008-5472.CAN-08-3722 19258498 PMC3822436

[B60] Chen Y Cairns R Papandreou I Koong A Denko NC Oxygen consumption can regulate the growth of tumors, a new perspective on the warburg effect PLoS One 2009 4 e7033 10.1371/journal.pone.0007033 19753307 PMC2737639

[B61] Ramanathan A Wang C Schreiber SL Perturbational profiling of a cell-line model of tumorigenesis by using metabolic measurements Proc Natl Acad Sci USA 2005 102 5992 5997 10.1073/pnas.0502267102 15840712 PMC1087961

[B62] Mayevsky A Mitochondrial function and energy metabolism in cancer cells: Past overview and future perspectives Mitochondrion 2009 19460294 10.1016/j.mito.2009.01.009

[B63] van Wijk R Souren J Schamhart DH van Miltenburg JC Comparative studies of the heat production of different rat hepatoma cells in culture Cancer Res 1984 44 671 673 6692371

[B64] Smith AE Kenyon DH A unifying concept of carcinogenesis and its therapeutic implications Oncology 1973 27 459 479 10.1159/000224754 4578174

[B65] Yuneva M Finding an "Achilles' heel" of cancer: the role of glucose and glutamine metabolism in the survival of transformed cells Cell Cycle 2008 7 2083 2089 18635953 10.4161/cc.7.14.6256

[B66] Deberardinis RJ Cheng T Q's next: the diverse functions of glutamine in metabolism, cell biology and cancer Oncogene 2009 19881548 10.1038/onc.2009.358 PMC2809806

[B67] Yang C Sudderth J Dang T Bachoo RG McDonald JG Deberardinis RJ Glioblastoma Cells Require Glutamate Dehydrogenase to Survive Impairments of Glucose Metabolism or Akt Signaling Cancer Res 2009 10.1158/0008-5472.CAN-09-2266 PMC2764330 19826036

[B68] John AP Dysfunctional mitochondria, not oxygen insufficiency, cause cancer cells to produce inordinate amounts of lactic acid: the impact of this on the treatment of cancer Med Hypotheses 2001 57 429 431 10.1054/mehy.2001.1335 11601863

[B69] Galluzzi L Morselli E Kepp O Vitale I Rigoni A Vacchelli E Michaud M Zischka H Castedo M Kroemer G Mitochondrial gateways to cancer Mol Aspects Med 2009 19698742 10.1016/j.mam.2009.08.002

[B70] Foster CS Spoerri PE Glees P Spoerri O The mode of mitochondrial degeneration in gliomas Acta Neurochir (Wien) 1978 43 229 237 10.1007/BF01587958 707179

[B71] Rasmussen AK Chatterjee A Rasmussen LJ Singh KK Mitochondria-mediated nuclear mutator phenotype in Saccharomyces cerevisiae Nucleic Acids Res 2003 31 3909 3917 10.1093/nar/gkg446 12853606 PMC165961

[B72] Cuezva JM Krajewska M de Heredia ML Krajewski S Santamaria G Kim H Zapata JM Marusawa H Chamorro M Reed JC The bioenergetic signature of cancer: a marker of tumor progression Cancer Res 2002 62 6674 6681 12438266

[B73] Kiebish MA Han X Cheng H Chuang JH Seyfried TN Cardiolipin and electron transport chain abnormalities in mouse brain tumor mitochondria: Lipidomic evidence supporting the Warburg theory of cancer J Lipid Res 2008 18703489 10.1194/jlr.M800319-JLR200 PMC2582368

[B74] Arismendi-Morillo GJ Castellano-Ramirez AV Ultrastructural mitochondrial pathology in human astrocytic tumors: potentials implications pro-therapeutics strategies J Electron Microsc (Tokyo) 2008 57 33 39 10.1093/jmicro/dfm038 18230641

[B75] Kiebish MA Han X Cheng H Seyfried TN In vitro growth environment produces lipidomic and electron transport chain abnormalities in mitochondria from non-tumorigenic astrocytes and brain tumours ASN Neuro 2009 1 10.1042/AN20090011 PMC2695587 19570033

[B76] Diaz-Ruiz R Uribe-Carvajal S Devin A Rigoulet M Tumor cell energy metabolism and its common features with yeast metabolism Biochim Biophys Acta 2009 1796 252 265 19682552 10.1016/j.bbcan.2009.07.003

[B77] Crabtree HG Observations on the carbohydrate metabolism of tumors Biochem J 1929 23 536 545 16744238 10.1042/bj0230536 PMC1254097

[B78] Bellance N Benard G Furt F Begueret H Smolkova K Passerieux E Delage JP Baste JM Moreau P Rossignol R Bioenergetics of lung tumors: alteration of mitochondrial biogenesis and respiratory capacity Int J Biochem Cell Biol 2009 41 2566 2577 10.1016/j.biocel.2009.08.012 19712747

[B79] Jiang F Ryan MT Schlame M Zhao M Gu Z Klingenberg M Pfanner N Greenberg ML Absence of cardiolipin in the crd1 null mutant results in decreased mitochondrial membrane potential and reduced mitochondrial function J Biol Chem 2000 275 22387 22394 10.1074/jbc.M909868199 10777514

[B80] Claypool SM Oktay Y Boontheung P Loo JA Koehler CM Cardiolipin defines the interactome of the major ADP/ATP carrier protein of the mitochondrial inner membrane J Cell Biol 2008 182 937 950 10.1083/jcb.200801152 18779372 PMC2528576

[B81] Ohtsuka T Nishijima M Suzuki K Akamatsu Y Mitochondrial dysfunction of a cultured Chinese hamster ovary cell mutant deficient in cardiolipin J Biol Chem 1993 268 22914 22919 8226801

[B82] Chicco AJ Sparagna GC Role of cardiolipin alterations in mitochondrial dysfunction and disease American journal of physiology Cell physiology 2007 292 C33 44 10.1152/ajpcell.00243.2006 16899548

[B83] Schug ZT Gottlieb E Cardiolipin acts as a mitochondrial signalling platform to launch apoptosis Biochim Biophys Acta 2009 19450542 10.1016/j.bbamem.2009.05.004

[B84] Veech RL The therapeutic implications of ketone bodies: the effects of ketone bodies in pathological conditions: ketosis, ketogenic diet, redox states, insulin resistance, and mitochondrial metabolism Prostaglandins Leukot Essent Fatty Acids 2004 70 309 319 10.1016/j.plefa.2003.09.007 14769489

[B85] Trachootham D Alexandre J Huang P Targeting cancer cells by ROS-mediated mechanisms: a radical therapeutic approach? Nat Rev Drug Discov 2009 8 579 591 10.1038/nrd2803 19478820

[B86] Detmer SA Chan DC Functions and dysfunctions of mitochondrial dynamics Nat Rev Mol Cell Biol 2007 8 870 879 10.1038/nrm2275 17928812

[B87] Acebo P Giner D Calvo P Blanco-Rivero A Ortega AD Fernandez PL Roncador G Fernandez-Malave E Chamorro M Cuezva JM Cancer abolishes the tissue type-specific differences in the phenotype of energetic metabolism Transl Oncol 2009 2 138 145 19701498 10.1593/tlo.09106 PMC2730139

[B88] Unwin RD Craven RA Harnden P Hanrahan S Totty N Knowles M Eardley I Selby PJ Banks RE Proteomic changes in renal cancer and co-ordinate demonstration of both the glycolytic and mitochondrial aspects of the Warburg effect Proteomics 2003 3 1620 1632 10.1002/pmic.200300464 12923786

[B89] Simonnet H Alazard N Pfeiffer K Gallou C Beroud C Demont J Bouvier R Schagger H Godinot C Low mitochondrial respiratory chain content correlates with tumor aggressiveness in renal cell carcinoma Carcinogenesis 2002 23 759 768 10.1093/carcin/23.5.759 12016148

[B90] Roskelley RC Mayer N Horwitt BN Salter WT Studies in Cancer. Vii. Enzyme Deficiency in Human and Experimental Cancer J Clin Invest 1943 22 743 751 10.1172/JCI101447 16695058 PMC435291

[B91] Rasnick D Duesberg PH How aneuploidy affects metabolic control and causes cancer Biochem J 1999 340 Pt 3 621 630 10.1042/0264-6021:3400621 10359645 PMC1220292

[B92] Parsons DW Jones S Zhang X Lin JC Leary RJ Angenendt P Mankoo P Carter H Siu IM Gallia GL Olivi A McLendon R Rasheed BA Keir S Nikolskaya T Nikolsky Y Busam DA Tekleab H Diaz LA Jr Hartigan J Smith DR Strausberg RL Marie SK Shinjo SM Yan H Riggins GJ Bigner DD Karchin R Papadopoulos N Parmigiani G Vogelstein B Velculescu VE Kinzler KW An integrated genomic analysis of human glioblastoma multiforme Science 2008 321 1807 1812 10.1126/science.1164382 18772396 PMC2820389

[B93] Jones S Zhang X Parsons DW Lin JC Leary RJ Angenendt P Mankoo P Carter H Kamiyama H Jimeno A Hong SM Fu B Lin MT Calhoun ES Kamiyama M Walter K Nikolskaya T Nikolsky Y Hartigan J Smith DR Hidalgo M Leach SD Klein AP Jaffee EM Goggins M Maitra A Iacobuzio-Donahue C Eshleman JR Kern SE Hruban RH Karchin R Papadopoulos N Parmigiani G Vogelstein B Velculescu VE Kinzler KW Core signaling pathways in human pancreatic cancers revealed by global genomic analyses Science 2008 321 1801 1806 10.1126/science.1164368 18772397 PMC2848990

[B94] Pollard PJ Wortham NC Tomlinson IP The TCA cycle and tumorigenesis: the examples of fumarate hydratase and succinate dehydrogenase Ann Med 2003 35 632 639 10.1080/07853890310018458 14708972

[B95] Hao HX Khalimonchuk O Schraders M Dephoure N Bayley JP Kunst H Devilee P Cremers CW Schiffman JD Bentz BG Gygi SP Winge DR Kremer H Rutter J SDH5, a Gene Required for Flavination of Succinate Dehydrogenase, Is Mutated in Paraganglioma Science 2009 19628817 10.1126/science.1175689 PMC3881419

[B96] Baysal BE Ferrell RE Willett-Brozick JE Lawrence EC Myssiorek D Bosch A Mey A van der Taschner PE Rubinstein WS Myers EN Richard CW Cornelisse CJ Devilee P Devlin B Mutations in SDHD, a mitochondrial complex II gene, in hereditary paraganglioma Science 2000 287 848 851 10.1126/science.287.5454.848 10657297

[B97] Alam NA Rowan AJ Wortham NC Pollard PJ Mitchell M Tyrer JP Barclay E Calonje E Manek S Adams SJ Bowers PW Burrows NP Charles-Holmes R Cook LJ Daly BM Ford GP Fuller LC Hadfield-Jones SE Hardwick N Highet AS Keefe M MacDonald-Hull SP Potts ED Crone M Wilkinson S Camacho-Martinez F Jablonska S Ratnavel R MacDonald A Mann RJ Grice K Guillet G Lewis-Jones MS McGrath H Seukeran DC Morrison PJ Fleming S Rahman S Kelsell D Leigh I Olpin S Tomlinson IP Genetic and functional analyses of FH mutations in multiple cutaneous and uterine leiomyomatosis, hereditary leiomyomatosis and renal cancer, and fumarate hydratase deficiency Hum Mol Genet 2003 12 1241 1252 10.1093/hmg/ddg148 12761039

[B98] Favier J Briere JJ Burnichon N Riviere J Vescovo L Benit P Giscos-Douriez I De Reynies A Bertherat J Badoual C Tissier F Amar L Libe R Plouin PF Jeunemaitre X Rustin P Gimenez-Roqueplo AP The warburg effect is genetically determined in inherited pheochromocytomas PLoS One 2009 4 e7094 10.1371/journal.pone.0007094 19763184 PMC2738974

[B99] Malkin D Li FP Strong LC Fraumeni JF Jr Nelson CE Kim DH Kassel J Gryka MA Bischoff FZ Tainsky MA Germ line p53 mutations in a familial syndrome of breast cancer, sarcomas, and other neoplasms Science 1990 250 1233 1238 10.1126/science.1978757 1978757

[B100] Yokota J Tumor progression and metastasis Carcinogenesis 2000 21 497 503 10.1093/carcin/21.3.497 10688870

[B101] Duesberg P Rasnick D Li R Winters L Rausch C Hehlmann R How aneuploidy may cause cancer and genetic instability Anticancer Res 1999 19 4887 4906 10697602

[B102] Kruse JP Gu W Modes of p53 regulation Cell 2009 137 609 622 10.1016/j.cell.2009.04.050 19450511 PMC3737742

[B103] Olovnikov IA Kravchenko JE Chumakov PM Homeostatic functions of the p53 tumor suppressor: regulation of energy metabolism and antioxidant defense Semin Cancer Biol 2009 19 32 41 10.1016/j.semcancer.2008.11.005 19101635 PMC2646792

[B104] Sonnenschein C Soto AM Somatic mutation theory of carcinogenesis: why it should be dropped and replaced Mol Carcinog 2000 29 205 211 10.1002/1098-2744(200012)29:4<205::AID-MC1002>3.0.CO;2-W 11170258

[B105] Dang L White DW Gross S Bennett BD Bittinger MA Driggers EM Fantin VR Jang HG Jin S Keenan MC Marks KM Prins RM Ward PS Yen KE Liau LM Rabinowitz JD Cantley LC Thompson CB Heiden MG Vander Su SM Cancer-associated IDH1 mutations produce 2-hydroxyglutarate Nature 2009 19935646 10.1038/nature08617 PMC2818760

[B106] Traven A Wong JM Xu D Sopta M Ingles CJ Interorganellar communication. Altered nuclear gene expression profiles in a yeast mitochondrial dna mutant J Biol Chem 2001 276 4020 4027 10.1074/jbc.M006807200 11054416

[B107] Veatch JR McMurray MA Nelson ZW Gottschling DE Mitochondrial dysfunction leads to nuclear genome instability via an iron-sulfur cluster defect Cell 2009 137 1247 1258 10.1016/j.cell.2009.04.014 19563757 PMC2759275

[B108] Jazwinski SM The retrograde response links metabolism with stress responses, chromatin-dependent gene activation, and genome stability in yeast aging Gene 2005 354 22 27 10.1016/j.gene.2005.03.040 15890475

[B109] Erol A Retrograde regulation due to mitochondrial dysfunction may be an important mechanism for carcinogenesis Med Hypotheses 2005 65 525 529 10.1016/j.mehy.2005.03.022 15905043

[B110] Butow RA Avadhani NG Mitochondrial signaling: the retrograde response Mol Cell 2004 14 1 15 10.1016/S1097-2765(04)00179-0 15068799

[B111] Amuthan G Biswas G Ananadatheerthavarada HK Vijayasarathy C Shephard HM Avadhani NG Mitochondrial stress-induced calcium signaling, phenotypic changes and invasive behavior in human lung carcinoma A549 cells Oncogene 2002 21 7839 7849 10.1038/sj.onc.1205983 12420221

[B112] Miceli MV Jazwinski SM Common and cell type-specific responses of human cells to mitochondrial dysfunction Exp Cell Res 2005 302 270 280 10.1016/j.yexcr.2004.09.006 15561107

[B113] Singh KK Kulawiec M Still I Desouki MM Geradts J Matsui S Inter-genomic cross talk between mitochondria and the nucleus plays an important role in tumorigenesis Gene 2005 354 140 146 10.1016/j.gene.2005.03.027 15979824

[B114] Liu Z Butow RA Mitochondrial retrograde signaling Annu Rev Genet 2006 40 159 185 10.1146/annurev.genet.40.110405.090613 16771627

[B115] Miceli MV Jazwinski SM Nuclear gene expression changes due to mitochondrial dysfunction in ARPE-19 cells: implications for age-related macular degeneration Invest Ophthalmol Vis Sci 2005 46 1765 1773 10.1167/iovs.04-1327 15851580

[B116] Kulawiec M Ayyasamy V Singh KK p53 regulates mtDNA copy number and mitocheckpoint pathway J Carcinog 2009 8 8 10.4103/1477-3163.50893 19439913 PMC2687143

[B117] Kulawiec M Safina A Desouki MM Still I Matsui SI Bakin A Singh KK Tumorigenic transformation of human breast epithelial cells induced by mitochondrial DNA depletion Cancer Biol Ther 2008 7 10.4161/cbt.7.11.6729 PMC2783327 19151587

[B118] Wolfman JC Planchon SM Liao J Wolfman A Structural and functional consequences of c-N-Ras constitutively associated with intact mitochondria Biochim Biophys Acta 2006 1763 1108 1124 10.1016/j.bbamcr.2006.07.015 16996152

[B119] Borghouts C Benguria A Wawryn J Jazwinski SM Rtg2 protein links metabolism and genome stability in yeast longevity Genetics 2004 166 765 777 10.1534/genetics.166.2.765 15020466 PMC1470750

[B120] Simbula G Glascott PA Jr Akita S Hoek JB Farber JL Two mechanisms by which ATP depletion potentiates induction of the mitochondrial permeability transition Am J Physiol 1997 273 C479 488 9277345 10.1152/ajpcell.1997.273.2.C479

[B121] Arnould T Vankoningsloo S Renard P Houbion A Ninane N Demazy C Remacle J Raes M CREB activation induced by mitochondrial dysfunction is a new signaling pathway that impairs cell proliferation Embo J 2002 21 53 63 10.1093/emboj/21.1.53 11782425 PMC125809

[B122] Whitfield JF Calcium, calcium-sensing receptor and colon cancer Cancer Lett 2009 275 9 16 10.1016/j.canlet.2008.07.001 18725175

[B123] Coussens LM Werb Z Inflammation and cancer Nature 2002 420 860 867 10.1038/nature01322 12490959 PMC2803035

[B124] Colotta F Allavena P Sica A Garlanda C Mantovani A Cancer-related inflammation, the seventh hallmark of cancer: links to genetic instability Carcinogenesis 2009 30 1073 1081 10.1093/carcin/bgp127 19468060

[B125] Amuthan G Biswas G Zhang SY Klein-Szanto A Vijayasarathy C Avadhani NG Mitochondria-to-nucleus stress signaling induces phenotypic changes, tumor progression and cell invasion Embo J 2001 20 1910 1920 10.1093/emboj/20.8.1910 11296224 PMC125420

[B126] Biswas G Guha M Avadhani NG Mitochondria-to-nucleus stress signaling in mammalian cells: nature of nuclear gene targets, transcription regulation, and induced resistance to apoptosis Gene 2005 354 132 139 10.1016/j.gene.2005.03.028 15978749 PMC3800739

[B127] Semenza GL Oxygen-dependent regulation of mitochondrial respiration by hypoxia-inducible factor 1 Biochem J 2007 405 1 9 17555402 10.1042/BJ20070389

[B128] Dang CV Semenza GL Oncogenic alterations of metabolism Trends Biochem Sci 1999 24 68 72 10.1016/S0968-0004(98)01344-9 10098401

[B129] Denko NC Hypoxia, HIF1 and glucose metabolism in the solid tumour Nat Rev Cancer 2008 8 705 713 10.1038/nrc2468 19143055

[B130] Tennant DA Duran RV Boulahbel H Gottlieb E Metabolic transformation in cancer Carcinogenesis 2009 30 1269 1280 10.1093/carcin/bgp070 19321800

[B131] King A Selak MA Gottlieb E Succinate dehydrogenase and fumarate hydratase: linking mitochondrial dysfunction and cancer Oncogene 2006 25 4675 4682 10.1038/sj.onc.1209594 16892081

[B132] Rius J Guma M Schachtrup C Akassoglou K Zinkernagel AS Nizet V Johnson RS Haddad GG Karin M NF-kappaB links innate immunity to the hypoxic response through transcriptional regulation of HIF-1alpha Nature 2008 453 807 811 10.1038/nature06905 18432192 PMC2669289

[B133] Zhang L Li L Liu H Prabhakaran K Zhang X Borowitz JL Isom GE HIF-1alpha activation by a redox-sensitive pathway mediates cyanide-induced BNIP3 upregulation and mitochondrial-dependent cell death Free Radic Biol Med 2007 43 117 127 10.1016/j.freeradbiomed.2007.04.005 17561100 PMC2048659

[B134] Haeberle HA Durrstein C Rosenberger P Hosakote YM Kuhlicke J Kempf VA Garofalo RP Eltzschig HK Oxygen-independent stabilization of hypoxia inducible factor (HIF)-1 during RSV infection PLoS One 2008 3 e3352 10.1371/journal.pone.0003352 18839041 PMC2556398

[B135] Moon EJ Jeong CH Jeong JW Kim KR Yu DY Murakami S Kim CW Kim KW Hepatitis B virus × protein induces angiogenesis by stabilizing hypoxia-inducible factor-1alpha Faseb J 2004 18 382 384 14688211 10.1096/fj.03-0153fje

[B136] Dang CV Le A Gao P MYC-Induced Cancer Cell Energy Metabolism and Therapeutic Opportunities Clin Cancer Res 2009 19861459 10.1158/1078-0432.CCR-09-0889 PMC2783410

[B137] Kolodner RD Putnam CD Myung K Maintenance of genome stability in Saccharomyces cerevisiae Science 2002 297 552 557 10.1126/science.1075277 12142524

[B138] Delsite R Kachhap S Anbazhagan R Gabrielson E Singh KK Nuclear genes involved in mitochondria-to-nucleus communication in breast cancer cells Mol Cancer 2002 1 6 10.1186/1476-4598-1-6 12495447 PMC149409

[B139] Evans AR Limp-Foster M Kelley MR Going APE over ref-1 Mutat Res 2000 461 83 108 11018583 10.1016/s0921-8777(00)00046-x

[B140] Ma Y Bai RK Trieu R Wong LJ Mitochondrial dysfunction in human breast cancer cells and their transmitochondrial cybrids Biochim Biophys Acta 2010 1797 29 37 10.1016/j.bbabio.2009.07.008 19647716 PMC2787670

[B141] Lebedeva MA Eaton JS Shadel GS Loss of p53 causes mitochondrial DNA depletion and altered mitochondrial reactive oxygen species homeostasis Biochim Biophys Acta 2009 1787 328 334 10.1016/j.bbabio.2009.01.004 19413947 PMC2680458

[B142] Holley AK St Clair DK Watching the watcher: regulation of p53 by mitochondria Future Oncol 2009 5 117 130 10.2217/14796694.5.1.117 19243304 PMC2710969

[B143] Busso CS Iwakuma T Izumi T Ubiquitination of mammalian AP endonuclease (APE1) regulated by the p53-MDM2 signaling pathway Oncogene 2009 28 1616 1625 10.1038/onc.2009.5 19219073 PMC2664849

[B144] Burhans WC Heintz NH The cell cycle is a redox cycle: Linking phase-specific targets to cell fate Free Radic Biol Med 2009 19486941 10.1016/j.freeradbiomed.2009.05.026

[B145] Whitaker M Calcium microdomains and cell cycle control Cell Calcium 2006 40 585 592 10.1016/j.ceca.2006.08.018 17045645 PMC3292880

[B146] Liu Y Malureanu L Jeganathan KB Tran DD Lindquist LD van Deursen JM Bram RJ CAML loss causes anaphase failure and chromosome missegregation Cell Cycle 2009 8 940 949 19229138 10.4161/cc.8.6.7948 PMC2967022

[B147] Marx J Cell biology. Do centrosome abnormalities lead to cancer? Science 2001 292 426 429 10.1126/science.292.5516.426 11330289

[B148] Chang DC Meng C A localized elevation of cytosolic free calcium is associated with cytokinesis in the zebrafish embryo J Cell Biol 1995 131 1539 1545 10.1083/jcb.131.6.1539 8522610 PMC2120692

[B149] Salmon ED Segall RR Calcium-labile mitotic spindles isolated from sea urchin eggs (Lytechinus variegatus) J Cell Biol 1980 86 355 365 10.1083/jcb.86.2.355 7190569 PMC2111485

[B150] Anghileri LJ Warburg's cancer theory revisited: a fundamentally new approach Arch Geschwulstforsch 1983 53 1 8 6860083

[B151] Fosslien E Cancer morphogenesis: role of mitochondrial failure Ann Clin Lab Sci 2008 38 307 329 18988924

[B152] Parkin DM The global health burden of infection-associated cancers in the year 2002 Int J Cancer 2006 118 3030 3044 10.1002/ijc.21731 16404738

[B153] Koike K Hepatitis B virus X gene is implicated in liver carcinogenesis Cancer Lett 2009 19464104 10.1016/j.canlet.2009.04.010

[B154] Clippinger AJ Bouchard MJ Hepatitis B virus HBx protein localizes to mitochondria in primary rat hepatocytes and modulates mitochondrial membrane potential J Virol 2008 82 6798 6811 10.1128/JVI.00154-08 18448529 PMC2446973

[B155] D'Agostino DM Bernardi P Chieco-Bianchi L Ciminale V Mitochondria as functional targets of proteins coded by human tumor viruses Adv Cancer Res 2005 94 87 142 10.1016/S0065-230X(05)94003-7 16096000

[B156] Koura M Isaka H Yoshida MC Tosu M Sekiguchi T Suppression of tumorigenicity in interspecific reconstituted cells and cybrids Gann 1982 73 574 580 7152196

[B157] Israel BA Schaeffer WI Cytoplasmic suppression of malignancy In Vitro Cell Dev Biol 1987 23 627 632 10.1007/BF02621071 3654482

[B158] Howell AN Sager R Tumorigenicity and its suppression in cybrids of mouse and Chinese hamster cell lines Proc Natl Acad Sci USA 1978 75 2358 2362 10.1073/pnas.75.5.2358 276880 PMC392552

[B159] Petros JA Baumann AK Ruiz-Pesini E Amin MB Sun CQ Hall J Lim S Issa MM Flanders WD Hosseini SH Marshall FF Wallace DC mtDNA mutations increase tumorigenicity in prostate cancer Proc Natl Acad Sci USA 2005 102 719 724 10.1073/pnas.0408894102 15647368 PMC545582

[B160] Hochedlinger K Blelloch R Brennan C Yamada Y Kim M Chin L Jaenisch R Reprogramming of a melanoma genome by nuclear transplantation Genes Dev 2004 18 1875 1885 10.1101/gad.1213504 15289459 PMC517407

[B161] Li L Connelly MC Wetmore C Curran T Morgan JI Mouse embryos cloned from brain tumors Cancer Res 2003 63 2733 2736 12782575

[B162] McKinnell RG Deggins BA Labat DD Transplantation of pluripotential nuclei from triploid frog tumors Science 1969 165 394 396 10.1126/science.165.3891.394 5815255

[B163] Harris H The analysis of malignancy by cell fusion: the position in 1988 Cancer Res 1988 48 3302 3306 3370633

[B164] Kroemer G Pouyssegur J Tumor cell metabolism: cancer's Achilles' heel Cancer Cell 2008 13 472 482 10.1016/j.ccr.2008.05.005 18538731

[B165] Tzachanis D Boussiotis VA Tob, a member of the APRO family, regulates immunological quiescence and tumor suppression Cell Cycle 2009 8 1019 1025 19270514 10.4161/cc.8.7.8033 PMC2908893

[B166] Sonnenschein C Soto AM The Society of Cells: Cancer and the Control of Cell Proliferation 1999 New York, Springer-Verlag

[B167] Godinot C de Laplanche E Hervouet E Simonnet H Actuality of Warburg's views in our understanding of renal cancer metabolism J Bioenerg Biomembr 2007 39 235 241 10.1007/s10863-007-9088-8 17665292

[B168] Marsh J Mukherjee P Seyfried TN Akt-dependent proapoptotic effects of dietary restriction on late-stage management of a phosphatase and tensin homologue/tuberous sclerosis complex 2-deficient mouse astrocytoma Clin Cancer Res 2008 14 7751 7762 10.1158/1078-0432.CCR-08-0213 19047102

[B169] Gao P Tchernyshyov I Chang TC Lee YS Kita K Ochi T Zeller KI De Marzo AM Van Eyk JE Mendell JT Dang CV c-Myc suppression of miR-23a/b enhances mitochondrial glutaminase expression and glutamine metabolism Nature 2009 458 762 765 10.1038/nature07823 19219026 PMC2729443

[B170] Wise DR DeBerardinis RJ Mancuso A Sayed N Zhang XY Pfeiffer HK Nissim I Daikhin E Yudkoff M McMahon SB Thompson CB Myc regulates a transcriptional program that stimulates mitochondrial glutaminolysis and leads to glutamine addiction Proc Natl Acad Sci USA 2008 105 18782 18787 10.1073/pnas.0810199105 19033189 PMC2596212

[B171] Bagheri S Nosrati M Li S Fong S Torabian S Rangel J Moore DH Federman S Laposa RR Baehner FL Sagebiel RW Cleaver JE Haqq C Debs RJ Blackburn EH Kashani-Sabet M Genes and pathways downstream of telomerase in melanoma metastasis Proc Natl Acad Sci USA 2006 103 11306 11311 10.1073/pnas.0510085103 16847266 PMC1544082

[B172] Saretzki G Telomerase, mitochondria and oxidative stress Exp Gerontol 2009 44 485 492 10.1016/j.exger.2009.05.004 19457450

[B173] Santos JH Meyer JN Van Houten B Mitochondrial localization of telomerase as a determinant for hydrogen peroxide-induced mitochondrial DNA damage and apoptosis Hum Mol Genet 2006 15 1757 1768 10.1093/hmg/ddl098 16613901

[B174] Ahmed S Passos JF Birket MJ Beckmann T Brings S Peters H Birch-Machin MA von Zglinicki T Saretzki G Telomerase does not counteract telomere shortening but protects mitochondrial function under oxidative stress J Cell Sci 2008 121 1046 1053 10.1242/jcs.019372 18334557

[B175] Mukherjee P Abate LE Seyfried TN Antiangiogenic and proapoptotic effects of dietary restriction on experimental mouse and human brain tumors Clin Cancer Res 2004 10 5622 5629 10.1158/1078-0432.CCR-04-0308 15328205

[B176] Mukherjee P El-Abbadi MM Kasperzyk JL Ranes MK Seyfried TN Dietary restriction reduces angiogenesis and growth in an orthotopic mouse brain tumour model Br J Cancer 2002 86 1615 1621 10.1038/sj.bjc.6600298 12085212 PMC2746602

[B177] Iruela-Arispe ML Dvorak HF Angiogenesis: a dynamic balance of stimulators and inhibitors Thromb Haemost 1997 78 672 677 9198237

[B178] Folkman J The role of angiogenesis in tumor growth Semin Cancer Biol 1992 3 65 71 1378311

[B179] Folkman J Incipient angiogenesis J Natl Cancer Inst 2000 92 94 95 10.1093/jnci/92.2.94 10639502

[B180] Greenberg JI Cheresh DA VEGF as an inhibitor of tumor vessel maturation: implications for cancer therapy Expert Opin Biol Ther 2009 9 1347 1356 10.1517/14712590903208883 19761418

[B181] Claffey KP Brown LF del Aguila LF Tognazzi K Yeo KT Manseau EJ Dvorak HF Expression of vascular permeability factor/vascular endothelial growth factor by melanoma cells increases tumor growth, angiogenesis, and experimental metastasis Cancer Res 1996 56 172 181 8548760

[B182] Ferrara N Gerber HP LeCouter J The biology of VEGF and its receptors Nat Med 2003 9 669 676 10.1038/nm0603-669 12778165

[B183] Bos R van Diest PJ de Jong JS Groep P van der Valk P van der Wall E van der Hypoxia-inducible factor-1alpha is associated with angiogenesis, and expression of bFGF, PDGF-BB, and EGFR in invasive breast cancer Histopathology 2005 46 31 36 10.1111/j.1365-2559.2005.02045.x 15656883

[B184] Tarin D Comparisons of metastases in different organs: biological and clinical implications Clin Cancer Res 2008 14 1923 1925 10.1158/1078-0432.CCR-07-5259 18381928

[B185] Bacac M Stamenkovic I Metastatic cancer cell Annu Rev Pathol 2008 3 221 247 10.1146/annurev.pathmechdis.3.121806.151523 18233952

[B186] Duffy MJ McGowan PM Gallagher WM Cancer invasion and metastasis: changing views J Pathol 2008 214 283 293 10.1002/path.2282 18095256

[B187] Steeg PS Tumor metastasis: mechanistic insights and clinical challenges Nat Med 2006 12 895 904 10.1038/nm1469 16892035

[B188] Chambers AF Groom AC MacDonald IC Dissemination and growth of cancer cells in metastatic sites Nat Rev Cancer 2002 2 563 572 10.1038/nrc865 12154349

[B189] Fidler IJ The pathogenesis of cancer metastasis: the 'seed and soil' hypothesis revisited Nat Rev Cancer 2003 3 453 458 10.1038/nrc1098 12778135

[B190] Huysentruyt LC Shelton LM Seyfried TN Influence of methotrexate and cisplatin on tumor progression and survival in the VM mouse model of systemic metastatic cancer Int J Cancer 2010 126 65 72 10.1002/ijc.24649 19536778

[B191] Khanna C Hunter K Modeling metastasis in vivo Carcinogenesis 2005 26 513 523 10.1093/carcin/bgh261 15358632

[B192] Steeg PS Heterogeneity of drug target expression among metastatic lesions: lessons from a breast cancer autopsy program Clin Cancer Res 2008 14 3643 3645 10.1158/1078-0432.CCR-08-1135 18559575 PMC2692037

[B193] Pawelek JM Cancer-cell fusion with migratory bone-marrow-derived cells as an explanation for metastasis: new therapeutic paradigms Future Oncol 2008 4 449 452 10.2217/14796694.4.4.449 18684055

[B194] Kalluri R EMT: when epithelial cells decide to become mesenchymal-like cells J Clin Invest 2009 119 1417 1419 10.1172/JCI39675 19487817 PMC2689122

[B195] Nowell PC The clonal evolution of tumor cell populations Science 1976 194 23 28 10.1126/science.959840 959840

[B196] Fearon ER Vogelstein B A genetic model for colorectal tumorigenesis Cell 1990 61 759 767 10.1016/0092-8674(90)90186-I 2188735

[B197] Carro MS Lim WK Alvarez MJ Bollo RJ Zhao X Snyder EY Sulman EP Anne SL Doetsch F Colman H Lasorella A Aldape K Califano A Iavarone A The transcriptional network for mesenchymal transformation of brain tumours Nature 1999 10.1038/nature08712 PMC4011561 20032975

[B198] Hart IR New evidence for tumour embolism as a mode of metastasis J Pathol 2009 219 275 276 10.1002/path.2616 19768739

[B199] Garber K Epithelial-to-mesenchymal transition is important to metastasis, but questions remain J Natl Cancer Inst 2008 100 232 233 10.1093/jnci/djn032 18270330

[B200] Lu X Kang Y Cell Fusion as a Hidden Force in Tumor Progression Cancer Res 2009 10.1158/0008-5472.CAN-09-2159 PMC2783941 19887616

[B201] Munzarova M Lauerova L Kovarik J Rejthar A Brezina V Kellnerova R Kovarik A Fusion-induced malignancy?. A preliminary study. (a challenge to today's common wisdom) Neoplasma 1992 39 79 86 1528312

[B202] Ruff MR Pert CB Small cell carcinoma of the lung: macrophage-specific antigens suggest hemopoietic stem cell origin Science 1984 225 1034 1036 10.1126/science.6089338 6089338

[B203] Fais S Cannibalism: a way to feed on metastatic tumors Cancer Lett 2007 258 155 164 10.1016/j.canlet.2007.09.014 17977647

[B204] Huysentruyt LC Mukherjee P Banerjee D Shelton LM Seyfried TN Metastatic cancer cells with macrophage properties: evidence from a new murine tumor model Int J Cancer 2008 123 73 84 10.1002/ijc.23492 18398829

[B205] Munzarova M Kovarik J Is cancer a macrophage-mediated autoaggressive disease? Lancet 1987 1 952 954 10.1016/S0140-6736(87)90295-9 2882343

[B206] Pawelek JM Chakraborty AK Fusion of tumour cells with bone marrow-derived cells: a unifying explanation for metastasis Nat Rev Cancer 2008 8 377 386 10.1038/nrc2371 18385683

[B207] Pawelek JM Tumour-cell fusion as a source of myeloid traits in cancer Lancet Oncol 2005 6 988 993 10.1016/S1470-2045(05)70466-6 16321767

[B208] Psaila B Lyden D The metastatic niche: adapting the foreign soil Nat Rev Cancer 2009 9 285 293 10.1038/nrc2621 19308068 PMC3682494

[B209] Munzarova M Lauerova L Capkova J Are advanced malignant melanoma cells hybrids between melanocytes and macrophages? Melanoma Res 1992 2 127 129 1643432 10.1097/00008390-199207000-00008

[B210] Lugini L Matarrese P Tinari A Lozupone F Federici C Iessi E Gentile M Luciani F Parmiani G Rivoltini L Malorni W Fais S Cannibalism of live lymphocytes by human metastatic but not primary melanoma cells Cancer Res 2006 66 3629 3638 10.1158/0008-5472.CAN-05-3204 16585188

[B211] Willenbring H Bailey AS Foster M Akkari Y Dorrell C Olson S Finegold M Fleming WH Grompe M Myelomonocytic cells are sufficient for therapeutic cell fusion in liver Nat Med 2004 10 744 748 10.1038/nm1062 15195088

[B212] Glinsky GV Berezovska O Glinskii AB Microarray analysis identifies a death-from-cancer signature predicting therapy failure in patients with multiple types of cancer J Clin Invest 2005 115 1503 1521 10.1172/JCI23412 15931389 PMC1136989

[B213] Joyce JA Pollard JW Microenvironmental regulation of metastasis Nat Rev Cancer 2009 9 239 252 10.1038/nrc2618 19279573 PMC3251309

[B214] Gordon S Snyderman JIGaR Development and distribution of mononuclear phagocytes: Relevance to inflammation Inflammation: Basic Principles and Clinical Correlates 1999 New York: Lippincott Williams & Wilkins 35 48

[B215] Lewis CE Pollard JW Distinct role of macrophages in different tumor microenvironments Cancer Res 2006 66 605 612 10.1158/0008-5472.CAN-05-4005 16423985

[B216] Pollard JW Macrophages define the invasive microenvironment in breast cancer J Leukoc Biol 2008 84 623 630 10.1189/jlb.1107762 18467655 PMC2516896

[B217] Scherer HJ A critical review: The pathology of cerebral gliomas J Neurol Neuropsychiat 1940 3 147 177 10.1136/jnnp.3.2.147 PMC1088179 21610973

[B218] Leenstra S Das PK Troost D de Boer OJ Bosch DA Human malignant astrocytes express macrophage phenotype J Neuroimmunol 1995 56 17 25 10.1016/0165-5728(94)00128-B 7822478

[B219] Youness E Barlogie B Ahearn M Trujillo JM Tumor cell phagocytosis. Its occurrence in a patient with medulloblastoma Arch Pathol Lab Med 1980 104 651 653 6893661

[B220] Kumar PV Hosseinzadeh M Bedayat GR Cytologic findings of medulloblastoma in crush smears Acta Cytol 2001 45 542 546 11480716 10.1159/000327862

[B221] Shabo I Olsson H Sun XF Svanvik J Expression of the macrophage antigen CD163 in rectal cancer cells is associated with early local recurrence and reduced survival time Int J Cancer 2009 125 1826 1831 10.1002/ijc.24506 19582880

[B222] Handerson T Camp R Harigopal M Rimm D Pawelek J Beta1,6-branched oligosaccharides are increased in lymph node metastases and predict poor outcome in breast carcinoma Clin Cancer Res 2005 11 2969 2973 10.1158/1078-0432.CCR-04-2211 15837749

[B223] Abodief WT Dey P Al-Hattab O Cell cannibalism in ductal carcinoma of breast Cytopathology 2006 17 304 305 10.1111/j.1365-2303.2006.00326.x 16961662

[B224] Marin-Padilla M Erythrophagocytosis by epithelial cells of a breast carcinoma Cancer 1977 39 1085 1089 10.1002/1097-0142(197703)39:3<1085::AID-CNCR2820390312>3.0.CO;2-U 199342

[B225] Spivak JL Phagocytic tumour cells Scand J Haematol 1973 11 253 256 4128872 10.1111/j.1600-0609.1973.tb00126.x

[B226] Ruff MR Farrar WL Pert CB Interferon gamma and granulocyte/macrophage colony-stimulating factor inhibit growth and induce antigens characteristic of myeloid differentiation in small-cell lung cancer cell lines Proc Natl Acad Sci USA 1986 83 6613 6617 10.1073/pnas.83.17.6613 3018738 PMC386554

[B227] Molad Y Stark P Prokocimer M Joshua H Pinkhas J Sidi Y Hemophagocytosis by small cell lung carcinoma Am J Hematol 1991 36 154 156 10.1002/ajh.2830360218 1849348

[B228] Falini B Bucciarelli E Grignani F Martelli MF Erythrophagocytosis by undifferentiated lung carcinoma cells Cancer 1980 1140 1145 10.1002/1097-0142(19800901)46:5<1140::AID-CNCR2820460511>3.0.CO;2-B 7214298

[B229] DeSimone PA East R Powell RD Jr Phagocytic tumor cell activity in oat cell carcinoma of the lung Hum Pathol 1980 11 535 539 6253386

[B230] Pawelek JM Chakraborty AK The cancer cell--leukocyte fusion theory of metastasis Adv Cancer Res 2008 101 397 444 10.1016/S0065-230X(08)00410-7 19055949

[B231] Rachkovsky M Sodi S Chakraborty A Avissar Y Bolognia J McNiff JM Platt J Bermudes D Pawelek J Melanoma × macrophage hybrids with enhanced metastatic potential Clin Exp Metastasis 1998 16 299 312 10.1023/A:1006557228604 9626809

[B232] Monteagudo C Jorda E Carda C Illueca C Peydro A Llombart-Bosch A Erythrophagocytic tumour cells in melanoma and squamous cell carcinoma of the skin Histopathology 1997 31 367 373 10.1046/j.1365-2559.1997.2670867.x 9363453

[B233] Breier F Feldmann R Fellenz C Neuhold N Gschnait F Primary invasive signet-ring cell melanoma J Cutan Pathol 1999 26 533 536 10.1111/j.1600-0560.1999.tb01802.x 10599947

[B234] Lazar D Taban S Dema A Cornianu M Goldis A Ratiu I Sporea I Gastric cancer: the correlation between the clinicopathological factors and patients' survival (I) Rom J Morphol Embryol 2009 50 41 50 19221644

[B235] Sung CO Seo JW Kim KM Do IG Kim SW Park CK Clinical significance of signet-ring cells in colorectal mucinous adenocarcinoma Mod Pathol 2008 21 1533 1541 10.1038/modpathol.2008.170 18849918

[B236] Moonda A Fatteh S Metastatic colorectal carcinoma: an unusual presentation J Cutan Pathol 2009 36 64 66 10.1111/j.1600-0560.2008.01007.x 18715256

[B237] Schorlemmer HU Bosslet K Kern HF Sedlacek HH Similarities in function between pancreatic tumor cells and macrophages and their inhibition by murine monoclonal antibodies Behring Inst Mitt 1988 240 264 3408453

[B238] Khayyata S Basturk O Adsay NV Invasive micropapillary carcinomas of the ampullo-pancreatobiliary region and their association with tumor-infiltrating neutrophils Mod Pathol 2005 18 1504 1511 10.1038/modpathol.3800460 16007065

[B239] Kojima S Sekine H Fukui I Ohshima H Clinical significance of "cannibalism" in urinary cytology of bladder cancer Acta Cytol 1998 42 1365 1369 9850644 10.1159/000332169

[B240] Chetty R Cvijan D Giant (bizarre) cell variant of renal carcinoma Histopathology 1997 30 585 587 10.1046/j.1365-2559.1997.5560789.x 9205865

[B241] Yasunaga M Ohishi Y Nishimura I Tamiya S Iwasa A Takagi E Inoue T Yahata H Kobayashi H Wake N Tsuneyoshi M Ovarian undifferentiated carcinoma resembling giant cell carcinoma of the lung Pathol Int 2008 58 244 248 10.1111/j.1440-1827.2008.02218.x 18324918

[B242] Lee H Sodek KL Hwang Q Brown TJ Ringuette M Sodek J Phagocytosis of collagen by fibroblasts and invasive cancer cells is mediated by MT1-MMP Biochem Soc Trans 2007 35 704 706 10.1042/BST0350704 17635128

[B243] Tsoi WC Feng CS Hemophagocytosis by rhabdomyosarcoma cells in bone marrow Am J Hematol 1997 54 340 342 10.1002/(SICI)1096-8652(199704)54:4<340::AID-AJH17>3.0.CO;2-F 9092697

[B244] Etcubanas E Peiper S Stass S Green A Rhabdomyosarcoma, presenting as disseminated malignancy from an unknown primary site: a retrospective study of ten pediatric cases Med Pediatr Oncol 1989 17 39 44 10.1002/mpo.2950170108 2913473

[B245] Seyfried TN Perspectives on brain tumor formation involving macrophages, glia, and neural stem cells Perspect Biol Med 2001 44 263 282 10.1353/pbm.2001.0035 11370160

[B246] Kim SY Roh JL Yeo NK Kim JS Lee JH Choi SH Nam SY Combined 18F-fluorodeoxyglucose-positron emission tomography and computed tomography as a primary screening method for detecting second primary cancers and distant metastases in patients with head and neck cancer Ann Oncol 2007 18 1698 1703 10.1093/annonc/mdm270 17716985

[B247] Hursting SD Kari FW The anti-carcinogenic effects of dietary restriction: mechanisms and future directions Mutat Res 1999 443 235 249 10415442 10.1016/s1383-5742(99)00021-6

[B248] Jose DG Good RA Quantitative effects of nutritional protein and calorie deficiency upon immune responses to tumors in mice Cancer Res 1973 33 807 812 4633154

[B249] Wheatley KE Williams EA Smith NC Dillard A Park EY Nunez NP Hursting SD Lane MA Low-carbohydrate diet versus caloric restriction: effects on weight loss, hormones, and colon tumor growth in obese mice Nutr Cancer 2008 60 61 68 10.1080/01635580802393118 18444137

[B250] Mukherjee P Sotnikov AV Mangian HJ Zhou JR Visek WJ Clinton SK Energy intake and prostate tumor growth, angiogenesis, and vascular endothelial growth factor expression J Natl Cancer Inst 1999 91 512 523 10.1093/jnci/91.6.512 10088621

[B251] Kari FW Dunn SE French JE Barrett JC Roles for insulin-like growth factor-1 in mediating the anti-carcinogenic effects of caloric restriction J Nutr Health Aging 1999 3 92 101 10885804

[B252] Mavropoulos JC Buschemeyer WC Tewari AK Rokhfeld D Pollak M Zhao Y Febbo PG Cohen P Hwang D Devi G Demark-Wahnefried W Westman EC Peterson BL Pizzo SV Freedland SJ The effects of varying dietary carbohydrate and fat content on survival in a murine LNCaP prostate cancer xenograft model Cancer Prev Res (Phila Pa) 2009 2 557 565 19470786 10.1158/1940-6207.CAPR-08-0188 PMC3774034

[B253] Bonorden MJ Rogozina OP Kluczny CM Grossmann ME Grambsch PL Grande JP Perkins S Lokshin A Cleary MP Intermittent calorie restriction delays prostate tumor detection and increases survival time in TRAMP mice Nutr Cancer 2009 61 265 275 10.1080/01635580802419798 19235043

[B254] Thompson HJ McGinley JN Spoelstra NS Jiang W Zhu Z Wolfe P Effect of dietary energy restriction on vascular density during mammary carcinogenesis Cancer Res 2004 64 5643 5650 10.1158/0008-5472.CAN-04-0787 15313902

[B255] Kritchevsky D Caloric restriction and experimental carcinogenesis Toxicol Sci 1999 52 13 16 10630585 10.1093/toxsci/52.2.13

[B256] Tannenbaum A Homburger F Nutrition and cancer Physiopathology of Cancer 1959 NY: Paul B. Hober 517 562

[B257] Kalaany NY Sabatini DM Tumours with PI3K activation are resistant to dietary restriction Nature 2009 458 725 731 10.1038/nature07782 19279572 PMC2692085

[B258] Hursting SD Smith SM Lashinger LM Harvey AE Perkins SN Calories and carcinogenesis: lessons learned from 30 years of calorie restriction research Carcinogenesis 2010 31 83 89 10.1093/carcin/bgp280 19969554

[B259] Pelicano H Xu RH Du M Feng L Sasaki R Carew JS Hu Y Ramdas L Hu L Keating MJ Zhang W Plunkett W Huang P Mitochondrial respiration defects in cancer cells cause activation of Akt survival pathway through a redox-mediated mechanism J Cell Biol 2006 175 913 923 10.1083/jcb.200512100 17158952 PMC2064701

[B260] Young CD Anderson SM Sugar and fat - that's where it's at: metabolic changes in tumors Breast Cancer Res 2008 10 202 10.1186/bcr1852 18304378 PMC2374962

[B261] Thompson HJ Jiang W Zhu Z Mechanisms by which energy restriction inhibits carcinogenesis Adv Exp Med Biol 1999 470 77 84 10709676 10.1007/978-1-4615-4149-3_8

[B262] Thompson HJ Zhu Z Jiang W Dietary Energy Restriction in Breast Cancer Prevention Journal of mammary gland biology and neoplasia 2003 8 133 142 10.1023/A:1025743607445 14587868

[B263] Mukherjee P Zhau J-R Sotnikov AV Clinton SK Teicher BA Dietary and nutritional modulation of tumor angiogenesis Antiangiogenic Agents in Cancer Therapy 1999 Totowa, NJ: Humana Press 237 261

[B264] Thompson HJ Zhu Z Jiang W Identification of the apoptosis activation cascade induced in mammary carcinomas by energy restriction Cancer Res 2004 64 1541 1545 10.1158/0008-5472.CAN-03-3108 14973070

[B265] Zhu Z Jiang W McGinley J Wolfe P Thompson HJ Effects of dietary energy repletion and IGF-1 infusion on the inhibition of mammary carcinogenesis by dietary energy restriction Molecular carcinogenesis 2005 42 170 176 10.1002/mc.20071 15599926

[B266] Hagopian K Ramsey JJ Weindruch R Influence of age and caloric restriction on liver glycolytic enzyme activities and metabolite concentrations in mice Exp Gerontol 2003 38 253 266 10.1016/S0531-5565(02)00203-6 12581789

[B267] Lee CK Klopp RG Weindruch R Prolla TA Gene expression profile of aging and its retardation by caloric restriction Science 1999 285 1390 1393 10.1126/science.285.5432.1390 10464095

[B268] Lee CK Weindruch R Prolla TA Gene-expression profile of the ageing brain in mice Nat Genet 2000 25 294 297 10.1038/77046 10888876

[B269] Mantis JG Centeno NA Todorova MT McGowan R Seyfried TN Management of multifactorial idiopathic epilepsy in EL mice with caloric restriction and the ketogenic diet: role of glucose and ketone bodies Nutr Metab (Lond) 2004 1 11 10.1186/1743-7075-1-11 15507133 PMC529249

[B270] Mahoney LB Denny CA Seyfried TN Caloric restriction in C57BL/6J mice mimics therapeutic fasting in humans Lipids Health Dis 2006 5 13 10.1186/1476-511X-5-13 16709251 PMC1513228

[B271] Cahill GF Jr Starvation in man N Engl J Med 1970 282 668 675 4915800 10.1056/NEJM197003192821209

[B272] Cahill GF Jr Veech RL Ketoacids? Good medicine? Trans Am Clin Climatol Assoc 2003 114 149 161 discussion 162-143 12813917 PMC2194504

[B273] Zhou W Mukherjee P Kiebish MA Markis WT Mantis JG Seyfried TN The calorically restricted ketogenic diet, an effective alternative therapy for malignant brain cancer Nutr Metab (Lond) 2007 4 5 10.1186/1743-7075-4-5 17313687 PMC1819381

[B274] Fredericks M Ramsey RB 3-Oxo acid coenzyme A transferase activity in brain and tumors of the nervous system J Neurochem 1978 31 1529 1531 10.1111/j.1471-4159.1978.tb06581.x 298319

[B275] Tisdale MJ Brennan RA Loss of acetoacetate coenzyme A transferase activity in tumours of peripheral tissues Br J Cancer 1983 47 293 297 6130780 10.1038/bjc.1983.38 PMC2011283

[B276] Seyfried NT Kiebish M Mukherjee P Ray S Targeting energy metabolism in brain cancer with restricted diets Glioblastoma: Molecular Mechanisms of Pathogenesis and Current Therapeutic Strategies 2010 New York: Springer 341 363

[B277] Seyfried TN Kiebish M Mukherjee P Marsh J Targeting energy metabolism in brain cancer with calorically restricted ketogenic diets Epilepsia 2008 49 Suppl 8 114 116 10.1111/j.1528-1167.2008.01853.x 19049606

[B278] Seyfried TN Sanderson TM El-Abbadi MM McGowan R Mukherjee P Role of glucose and ketone bodies in the metabolic control of experimental brain cancer Br J Cancer 2003 89 1375 1382 10.1038/sj.bjc.6601269 14520474 PMC2394295

[B279] Seyfried TN Mukherjee P Meadows GG Anti-Angiogenic and Pro-Apoptotic Effects of Dietary Restriction in Experimental Brain Cancer: Role of Glucose and Ketone Bodies Integration/Interaction of Oncologic Growth. Cancer Growth and Progression 2005 15 New York: Kluwer Academic 259 270 full_text

[B280] Nebeling LC Miraldi F Shurin SB Lerner E Effects of a ketogenic diet on tumor metabolism and nutritional status in pediatric oncology patients: two case reports J Am Coll Nutr 1995 14 202 208 7790697 10.1080/07315724.1995.10718495

[B281] Otto C Kaemmerer U Illert B Muehling B Pfetzer N Wittig R Voelker HU Thiede A Coy JF Growth of human gastric cancer cells in nude mice is delayed by a ketogenic diet supplemented with omega-3 fatty acids and medium-chain triglycerides BMC Cancer 2008 8 122 10.1186/1471-2407-8-122 18447912 PMC2408928

[B282] Mavropoulos JC Isaacs WB Pizzo SV Freedland SJ Is there a role for a low-carbohydrate ketogenic diet in the management of prostate cancer? Urology 2006 68 15 18 10.1016/j.urology.2006.03.073 16844447

[B283] Mukherjee P Mulrooney TJ Marsh J Blair D Chiles TC Seyfried TN Differential effects of energy stress on AMPK phosphorylation and apoptosis in experimental brain tumor and normal brain Mol Cancer 2008 7 37 10.1186/1476-4598-7-37 18474106 PMC2397440

[B284] Argiles JM Moore-Carrasco R Fuster G Busquets S Lopez-Soriano FJ Cancer cachexia: the molecular mechanisms Int J Biochem Cell Biol 2003 35 405 409 10.1016/S1357-2725(02)00251-0 12565701

[B285] Tisdale MJ Cancer anorexia and cachexia Nutrition 2001 17 438 442 10.1016/S0899-9007(01)00506-8 11377146

[B286] Todorov PT Wyke SM Tisdale MJ Identification and characterization of a membrane receptor for proteolysis-inducing factor on skeletal muscle Cancer Res 2007 67 11419 11427 10.1158/0008-5472.CAN-07-2602 18056470

[B287] Tisdale MJ Biology of cachexia J Natl Cancer Inst 1997 89 1763 1773 10.1093/jnci/89.23.1763 9392617

[B288] Lopez-Lazaro M The warburg effect: why and how do cancer cells activate glycolysis in the presence of oxygen? Anticancer Agents Med Chem 2008 8 305 312 10.2174/187152008783961932 18393789

[B289] Rodriguez-Enriquez S Marin-Hernandez A Gallardo-Perez JC Carreno-Fuentes L Moreno-Sanchez R Targeting of cancer energy metabolism Mol Nutr Food Res 2009 53 29 48 10.1002/mnfr.200700470 19123180

[B290] Pelicano H Martin DS Xu RH Huang P Glycolysis inhibition for anticancer treatment Oncogene 2006 25 4633 4646 10.1038/sj.onc.1209597 16892078

[B291] Marsh J Mukherjee P Seyfried TN Drug/diet synergy for managing malignant astrocytoma in mice: 2-deoxy-D-glucose and the restricted ketogenic diet Nutr Metab (Lond) 2008 5 33 10.1186/1743-7075-5-33 19032781 PMC2607273

[B292] Dvorak HF Tumors: wounds that do not heal. Similarities between tumor stroma generation and wound healing N Engl J Med 1986 315 1650 1659 3537791 10.1056/NEJM198612253152606

[B293] Dong W Selgrade MK Gilmour IM Lange RW Park P Luster MI Kari FW Altered alveolar macrophage function in calorie-restricted rats Am J Respir Cell Mol Biol 1998 19 462 469 9730874 10.1165/ajrcmb.19.3.3114

[B294] Medina MA Glutamine and cancer J Nutr 2001 131 2539S 2542S discussion 2550S-2531S 11533309 10.1093/jn/131.9.2539S

[B295] Newsholme P Why is L-glutamine metabolism important to cells of the immune system in health, postinjury, surgery or infection? J Nutr 2001 131 2515S 2522S discussion 2523S-2514S 11533304 10.1093/jn/131.9.2515S

[B296] Tijerina AJ The biochemical basis of metabolism in cancer cachexia Dimens Crit Care Nurs 2004 23 237 243 10.1097/00003465-200411000-00001 15586034

[B297] Piscitelli SC Thibault A Figg WD Tompkins A Headlee D Lieberman R Samid D Myers CE Disposition of phenylbutyrate and its metabolites, phenylacetate and phenylacetylglutamine J Clin Pharmacol 1995 35 368 373 7650225 10.1002/j.1552-4604.1995.tb04075.x

[B298] American Cancer Society Cancer Facts & Figures 2009 2009 American Cancer Society, Atlanta 68

[B299] Chance B Sies H Boveris A Hydroperoxide metabolism in mammalian organs Physiol Rev 1979 59 527 605 37532 10.1152/physrev.1979.59.3.527

[B300] Ziegler DR Ribeiro LC Hagenn M Siqueira IR Araujo E Torres IL Gottfried C Netto CA Goncalves CA Ketogenic diet increases glutathione peroxidase activity in rat hippocampus Neurochem Res 2003 28 1793 1797 10.1023/A:1026107405399 14649719

[B301] Elias SG Peeters PH Grobbee DE van Noord PA Breast cancer risk after caloric restriction during the 1944-1945 Dutch famine J Natl Cancer Inst 2004 96 539 546 15069116 10.1093/jnci/djh087

[B302] Hursting SD Forman MR Cancer risk from extreme stressors: lessons from European Jewish survivors of World War II J Natl Cancer Inst 2009 101 1436 1437 10.1093/jnci/djp357 19861304

[B303] Qiao YL Dawsey SM Kamangar F Fan JH Abnet CC Sun XD Johnson LL Gail MH Dong ZW Yu B Mark SD Taylor PR Total and cancer mortality after supplementation with vitamins and minerals: follow-up of the Linxian General Population Nutrition Intervention Trial J Natl Cancer Inst 2009 101 507 518 10.1093/jnci/djp037 19318634 PMC2664089

[B304] Balietti M Fattoretti P Giorgetti B Casoli T Di Stefano G Solazzi M Platano D Aicardi G Bertoni-Freddari C A ketogenic diet increases succinic dehydrogenase activity in aging cardiomyocytes Ann N Y Acad Sci 2009 1171 377 384 10.1111/j.1749-6632.2009.04704.x 19723079

[B305] Sato K Kashiwaya Y Keon CA Tsuchiya N King MT Radda GK Chance B Clarke K Veech RL Insulin, ketone bodies, and mitochondrial energy transduction Faseb J 1995 9 651 658 7768357 10.1096/fasebj.9.8.7768357

[B306] Cleary MP Jacobson MK Phillips FC Getzin SC Grande JP Maihle NJ Weight-cycling decreases incidence and increases latency of mammary tumors to a greater extent than does chronic caloric restriction in mouse mammary tumor virus-transforming growth factor-alpha female mice Cancer Epidemiol Biomarkers Prev 2002 11 836 843 12223427

[B307] Kritchevsky D Heber D, Blackburn GL, Go VLW Fundamentals of nutrition: applications to cancer research Nutritional Oncology 1999 Boston: Academic Press 5 10

[B308] Kritchevsky D Caloric restriction and experimental mammary carcinogenesis Breast Cancer Res Treat 1997 46 161 167 10.1023/A:1005960410225 9478271

[B309] Hopper BD Przybyszewski J Chen HW Hammer KD Birt DF Effect of ultraviolet B radiation on activator protein 1 constituent proteins and modulation by dietary energy restriction in SKH-1 mouse skin Mol Carcinog 2009 48 843 852 10.1002/mc.20529 19263438 PMC2736326

[B310] Steinbach G Heymsfield S Olansen NE Tighe A Holt PR Effect of caloric restriction on colonic proliferation in obese persons: implications for colon cancer prevention Cancer Res 1994 54 1194 1197 8118805

[B311] Albanes D Caloric intake, body weight, and cancer: a review Nutr Cancer 1987 9 199 217 10.1080/01635588709513929 3299283

